# Cdk2 strengthens the intra-S checkpoint and counteracts cell cycle exit induced by DNA damage

**DOI:** 10.1038/s41598-017-12868-5

**Published:** 2017-10-18

**Authors:** Katarina Bačević, Gérald Lossaint, Thiziri Nait Achour, Virginie Georget, Daniel Fisher, Vjekoslav Dulić

**Affiliations:** 1 IGMM, CNRS, Univ. Montpellier, Montpellier, France; 2 MRI-CRBM, CNRS, Univ. Montpellier, Montpellier, France; 3 Present Address: Swiss Institute for Experimental Cancer Research (ISREC), Ecole Polytechnique Fédérale de Lausanne (EPFL), 1015 Lausanne, Switzerland

## Abstract

Although cyclin-dependent kinase 2 (Cdk2) controls the G1/S transition and promotes DNA replication, it is dispensable for cell cycle progression due to redundancy with Cdk1. Yet Cdk2 also has non-redundant functions that can be revealed in certain genetic backgrounds and it was reported to promote the G2/M DNA damage response checkpoint in TP53 (p53)-deficient cancer cells. However, in p53-proficient cells subjected to DNA damage, Cdk2 is inactivated by the CDK inhibitor p21. We therefore investigated whether Cdk2 differentially affects checkpoint responses in p53-proficient and deficient cell lines. We show that, independently of p53 status, Cdk2 stimulates the ATR/Chk1 pathway and is required for an efficient DNA replication checkpoint response. In contrast, Cdk2 is not required for a sustained DNA damage response and G2 arrest. Rather, eliminating Cdk2 delays S/G2 progression after DNA damage and accelerates appearance of early markers of cell cycle exit. Notably, Cdk2 knockdown leads to down-regulation of Cdk6, which we show is a non-redundant pRb kinase whose elimination compromises cell cycle progression. Our data reinforce the notion that Cdk2 is a key p21 target in the DNA damage response whose inactivation promotes exit from the cell cycle in G2.

## Introduction

Cyclin-dependent kinase 2 (Cdk2) is a key cell cycle regulator, with roles in inactivating phosphorylation of the RB1 (pRb) tumour suppressor family and in controlling both G1/S and G2/M transitions. However, genetic invalidation or knock-down experiments have shown that, unlike Cdk1^1^, Cdk2 is dispensable for cell proliferation^2,3^, and is dispensable for much of mouse development^4–6^. This is due to functional redundancy with Cdk1, which, in the absence of Cdk2, can phosphorylate pRb by binding to D-type cyclins, and can promote replication in complex with Cyclin E1 (CycE1) and Cyclin A (CycA)^1,2^. Chemical genetics experiments are not subjected to compensation mechanisms that might occur in genetic knockout studies, and a recent study using analogue-sensitive Cdk2 alleles showed that Cdk2 promotes G1/S progression after cell cycle entry from quiescence in low serum^7^. However, no such studies have yet addressed the reported function of Cdk2 in promoting the G2/M progression^8,9^.

In addition to promoting cell cycle progression, Cdk2 has been described to play a positive role in cell cycle arrest in the DNA damage response (DDR), in particular at the G2/M checkpoint. Although Cdk2, Cdk3, Cdk4 and Cdk6 are dispensable for DNA damage checkpoints in MEFs^10^, several studies have reported that activation of the ATR-Chk1 pathway is impaired in the absence of Cdk2^11–14^. Moreover, in the absence of the p53-p21 pathway, Cdk2 appears to be essential for DNA damage-induced G2 arrest in HCT-116 colorectal cancer cells where, *via* stabilizing the DNA replication licensing protein Cdc6, it promotes activation of the ATR-Chk1 pathway^13^. Furthermore, a chemical genetics approach using analogue-sensitive alleles of Cdk2 identified that Cdk2 has a specific role in the DDR. Thus, Cdk2 inhibition apparently hinders the DDR, and sensitises cells to ionizing radiation, inducing cell death^15^. It was concluded that Cdk2 is required to arrest the cell cycle in response to ionizing radiation.

These results are difficult to reconcile with reports showing that most of CycA-Cdk2 complexes are bound to the CDK inhibitor p21 after triggering of the DDR in G2^16,17^, which rather suggest that Cdk2 inhibition is an integral part of the DDR. Additionally, Cdk2 suppresses c-myc-induced cellular senescence^18^, suggesting that Cdk2 inhibition may be required for cell cycle exit. If Cdk2 activity promotes the DNA damage response, why then should it be inhibited by p21? One possibility is that this switches off DNA replication in S-phase, while the major mechanism of action of p21 in the G2 arrest might be to inactivate CycB1-Cdk1 rather than Cdk2. While p21 has indeed been implicated in CycB1-Cdk1 inhibition^19–21^, it is dispensable for G2 arrest^22,23^.

To better understand the roles of Cdk2 in responses to replication stress and DNA damage, we studied both p53-proficient and p53-deficient cancer cells. We show that Cdk2 promotes Chk1 activation and cell cycle arrest induced by hydroxyurea. In contrast, Cdk2 is not required for Chk1 activation and G2 arrest by agents that induce double strand DNA breaks. On the contrary, ablation of Cdk2 strongly delays S-M progression upon DNA damage and down-regulates Cdk6. This leads to more rapid appearance of early markers of cell cycle exit. We propose that inhibition of Cdk2 by the DDR promotes a timely implementation of the G2 cell cycle exit programme.

## Results

### Cdk2 is required for efficient Chk1 activation and G1 arrest upon exposure to HU

**Figure 1:**
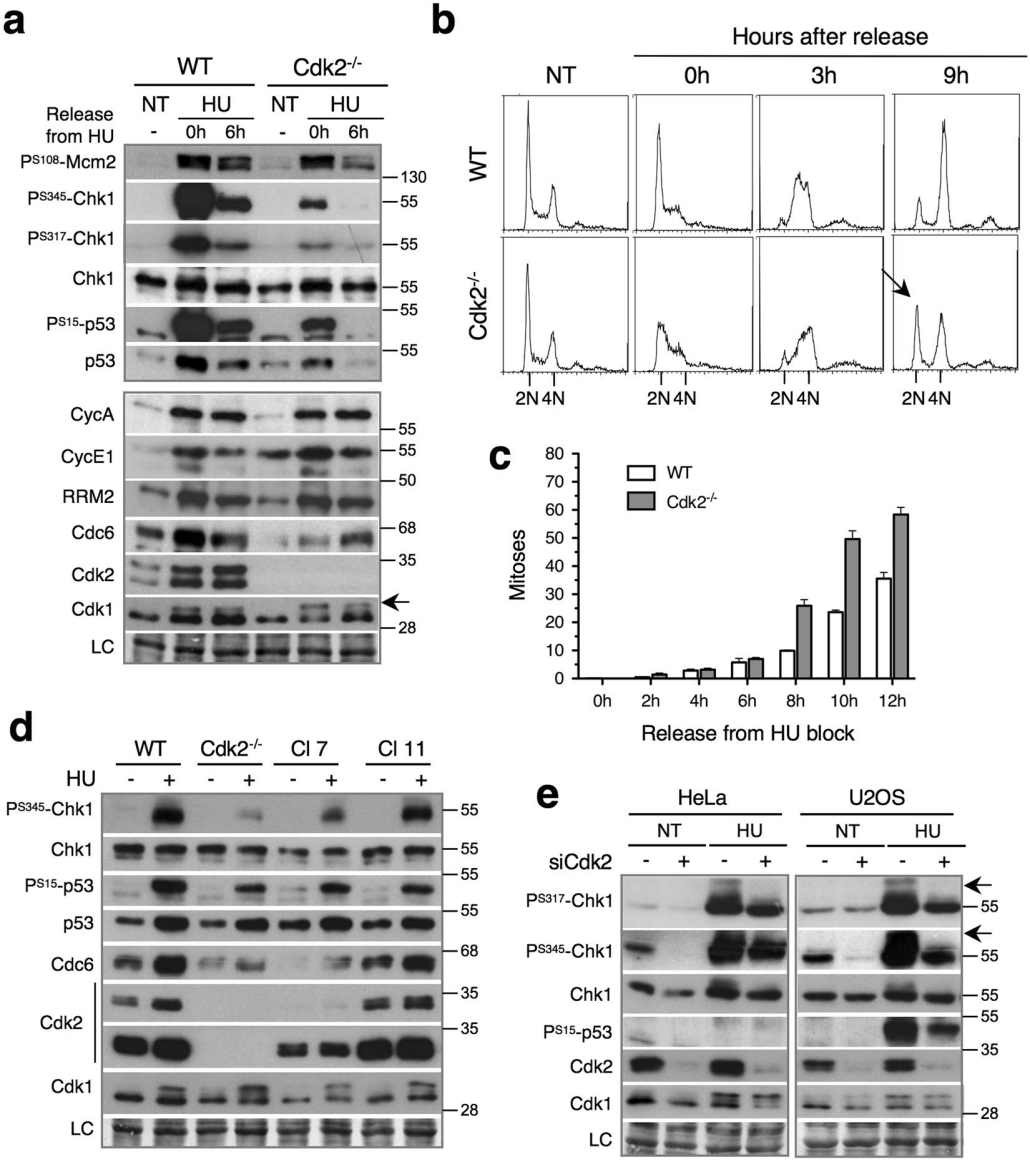
Cdk2 is required for efficient Chk1 activation and G1 arrest upon exposure to HU. (**a**) Immunoblots showing phosphorylation of Chk1, Mcm2 and p53 as well as the expression of indicated cell cycle regulators after 20 h in hydroxyurea (HU-0h) and 6 hrs after release, in wild-type (WT) and Cdk2^−/−^ HCT-116 cells. NT, non treated cells. Arrow shows hyper-phosphorylated Cdk1. LC, loading control. (**b**) Flow cytometry analysis showing cell cycle progression after release from HU block in WT and Cdk2^−/−^ HCT-116 cells. Arrows show accelerated appearance of G1 (2 N) cell population in Cdk2^−/−^ cells. See Supplementary Fig. S3 for quantification. (**c**) Video-microscopy data showing fraction of mitotic cells after the release of HU block in WT and Cdk2^−/−^ HCT-116 cells. Mean and standard deviation of three separate experiments are given. (**d**) Immunoblots showing phosphorylation of Chk1 and p53 as well as the expression of Cdc6 in the presence of 2 mM hydroxyurea (HU-0h, 20 h) in WT, Cdk2^−/−^ and two clones (clone 7 and 11) expressing Cdk2 in Cdk2^−/−^ HCT-116 cells. NT, non-treated cells. LC, loading control. (**e**) Immunoblots showing Chk1 phosphorylation in non-treated (NT) and HU-arrested control (Ctl) and Cdk2 knockdown (siCdk2) HeLa and U2OS cells. Arrows, phosphorylation-dependent SDS-PAGE mobility shift. LC, loading control. See Supplementary Fig. S4 for FACS analysis. Uncropped images of blots are shown in Supplementary Fig. S17.

Cdk2 is thought to promote cell cycle arrest by activating ATR-Chk1^12–14^. As the ATR-Chk1 pathway also controls the intra-S checkpoint, we first tested whether genetic ablation of Cdk2 in p53–proficient HCT-116 cells interfered with Chk1 activation in response to hydroxyurea (HU), a ribonucleotide reductase inhibitor that blocks DNA replication. Indeed, we found that Chk1 and p53 phosphorylation were strongly reduced in Cdk2^−/−^ cells (Fig. 1a). By contrast, ATR-dependent phosphorylation of Mcm2 (P^S108^), a component of pre-replicative complexes (pre-RC), was much less affected. Cdk2 ablation strongly diminished levels of Cdc6, another component of pre-replication complexes that has been implicated in ATR activation^13^. However, the levels of ribonucleotide reductase catalytic subunit (RRM2), whose phosphorylation by Cdks promotes its degradation, or the cyclins that regulate S-phase progression, Cyclin E1 (CycE1) or Cyclin A (CycA), were not affected (Fig. 1a). Similar results were obtained upon siRNA-mediated Cdk2 depletion (Supplementary Fig. S1). Moreover, and consistent with an impaired intra-S checkpoint, the G1/S arrest by HU in Cdk2^−/−^ cells was inefficient in comparison to WT cells. Upon the release of Cdk2^−/−^ cells from the HU block, Cdk1 phosphorylation (Fig. 1a, *arrow*) and mitotic entry were significantly accelerated (Fig. 1b,c and Supplementary Fig. S2). Re-expression of Cdk2 in Cdk2^*−/−*^ HCT-116 cells reversed this phenotype (Fig. 1d and Supplementary Fig. S3). Thus, Cdk2 stimulates Chk1 activation and promotes the replication stress response even in cells with functional p53.

To see if this is true in other cell lines, we depleted Cdk2 by siRNA in p53-deficient HeLa and p53-proficient U2OS cells, and subjected them to HU. Indeed, Chk1 phosphorylation was markedly reduced upon Cdk2 knockdown (KD), confirming a role for the latter in Chk1 activation (Fig. 1d). However, Cdk2 depletion did not significantly impair G1/S arrest by HU in HeLa and U2OS cells (Supplementary Fig. S4). This is likely due to residual Cdk2/Chk1 activity, since although Cdk2 depletion in HCT-116 cells diminished Chk1 phosphorylation, it had little effect on G1/S arrest by HU, unlike complete Cdk2 knockout (Supplementary Fig. S1).

We conclude that while acute Cdk2 ablation does not affect normal cell cycle progression or CycE1 or CycA expression, Cdk2 is required for efficient Chk1 activation and thus promotes cell cycle arrest in response to replication stress.

### CycA-Cdk1/2 complexes are major DNA damage-induced p21 targets in HCT-116 cells

Next, we asked whether Cdk2 might also play a positive role in responses to genotoxic agents that cause double strand DNA breaks. Although a previous report implied that Cdk2 is required for G2 arrest in response to γ-irradiation, this role could be revealed only in a p53^−/−^ genetic background^13^, perhaps because p53, via its effector, p21, might obviate Cdk2 function in G2 by directly inhibiting CycB1-Cdk1. However, our earlier results suggested that CycB1-Cdk1 complexes are probably not major p21 targets in DNA damage-induced G2 arrest in several p53-proficient cell lines, including U2OS and HCT-116^23^.

**Figure 2:**
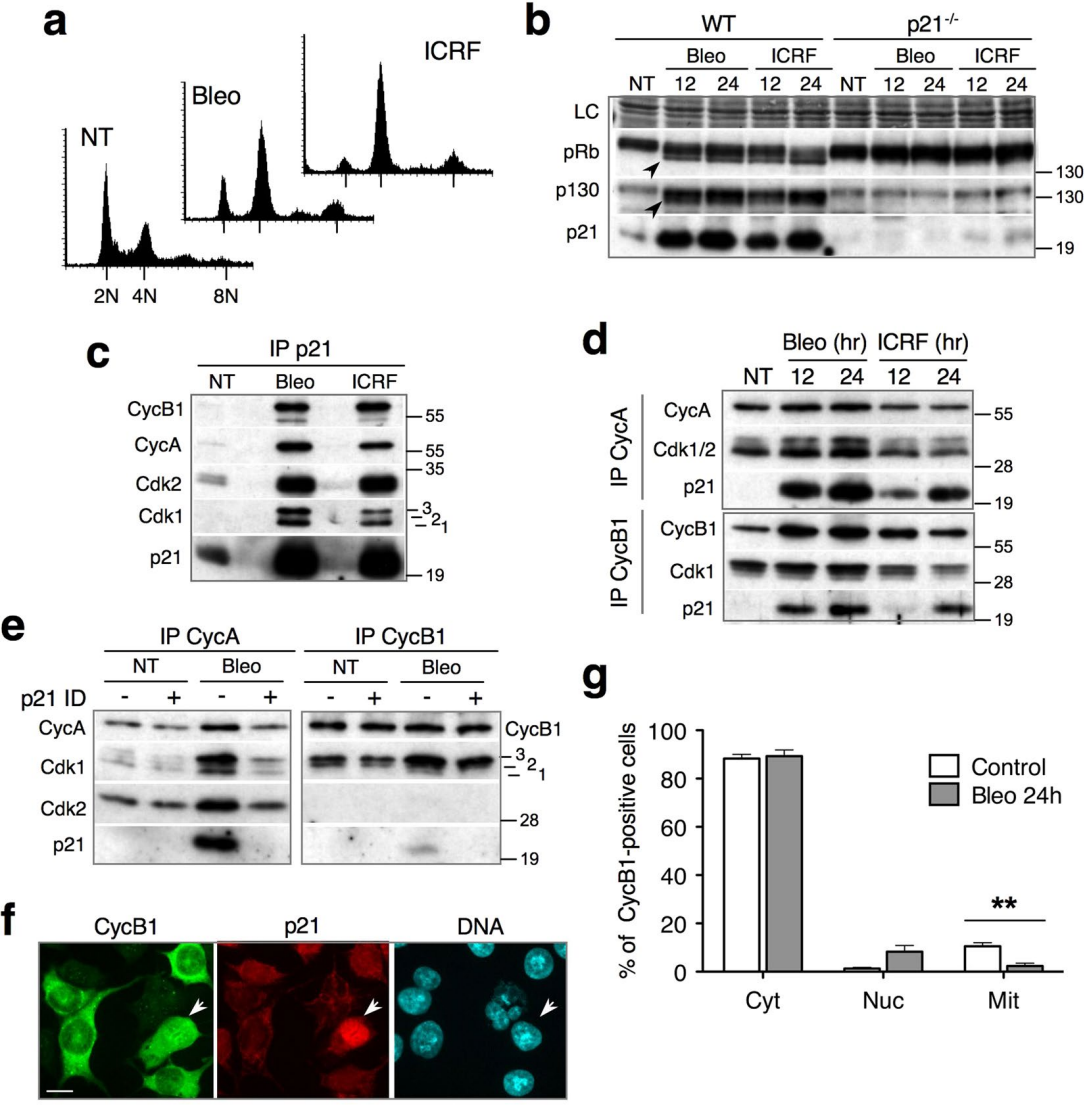
CycA-Cdk1/2 complexes are major DNA damage-induced p21 targets in HCT-116 cells. (**a**) Flow cytometry analysis of non-treated (NT) and HCT-116 cells exposed to bleomycin or ICRF-193 (ICRF) for 24 h. (**b**) Immunoblot analysis showing pRb and p130 pocket proteins in WT and p21^-/-^ HCT-116 cells exposed to bleomycin or ICRF for 12 and 24h. Arrows show hypo-phosphorylated pRb and p130. NT, non-treated cells. LC, loading control. (**c**) Immunoblot analysis of p21 immunoprecipitates (IP) from HCT-116 cells exposed to bleomycin or ICRF (24 h). Numbers 1, 2 and 3 indicate different Cdk1 phospho-isoforms. (**d**) Immunoblot analysis of CycA and CycB1 immunoprecipitates (IP) from HCT-116 cells exposed to bleomycin or ICRF for indicated times. (**e**) p21 immunodepletion (ID) experiment. Immunoblot analysis of CycA and CycB1 immunoprecipitates before (mock-treated: -) and after p21 immunodepletion (+) of cell extracts from non-treated (NT) or HCT-116 cells exposed to bleomycin or ICRF for 24 h. Numbers 1, 2 and 3 indicate different Cdk1 phospho-isoforms. (**f**) Immunofluorescence showing CycB1 and p21 localization in HCT-116 cells arrested in G2 with ICRF (24 h). Arrow shows a cell in G2 with high p21 content and nuclear CycB1. Bar, 10 mM. (**g**) CycB1 localization in non-treated (NT) and HCT-116 cells exposed to bleomycin (24 h). Cytoplasmic (Cyt), mainly or partly nuclear (Nuc) or mitotic (Mit) CycB1-positive cells were scored in five independent experiments (more than 1000 cells). Error bars indicate standard errors. Asterisks indicate that the effect of bleomycin is statistically significant (paired t-test) at the 0.01 level (p = 0.0089). Uncropped images of blots are shown in Supplementary Fig. S17.

We therefore assessed the contribution of p21 to inhibition of mitotic regulators in p53-proficient HCT-116 cells treated with a DNA topoisomerase II inhibitor, ICRF-193 (ICRF), or the radiomimetic drug bleomycin^17^. In these cells, both genotoxic agents induce G2 arrest (Fig. 2a), but ICRF-induced arrest is only transient and the cells progress quickly into mitosis (see below). In these conditions, p21 blocks the inactivating phosphorylation of pRb and the pRb family member p130 (Fig. 2b). Western blot analysis of immunoprecipitated p21 (Fig. 2c) and CycA and CycB1-associated Cdks (Fig. 2d) shows that the genotoxic agents induced p21 binding to both cyclin-Cdk complexes. Removal of p21-associated complexes by immunodepletion (ID) showed that p21 bound most CycA-Cdk1 and CycA-Cdk2 complexes but only a small fraction of CycB1-Cdk1 complexes (Fig. 2e). Consistent with these results, immunofluorescence analysis showed that, in G2-arrested HCT-116 cells, p21-dependent CycB1 nuclear localization was an extremely rare event (Fig. 2f,g and Supplementary Fig. S5). These results reveal that, in HCT-116 cells subjected to DNA damage, p21 does not directly inhibit CycB1-Cdk1 but preferentially targets CycA-Cdk1 and CycA-Cdk2 complexes. Taken together, these results suggest that inactivation of CycA-Cdk1/2 complexes by p21 might promote efficient G2 arrest and cell cycle exit.

### Cdk2 is not required for Chk1 activation upon DNA damage by bleomycin, and its absence slows S/G2 progression

The above results do not support the proposed roles of Cdk2 in promoting DNA damage-induced G2 arrest and Chk1 activation^13,15^. We thus further investigated the requirement for Cdk2 in Chk1 activation and DNA damage-induced cell cycle arrest. Since Cdk2 roles in this checkpoint were previously revealed in a p53^−/−^ background, we studied p53^−/−^ HCT-116 cells in addition to WT and Cdk2^−/−^ cells. We also investigated the role of Cdk2 in the DDR in U2OS cells, which are p53-proficient, and HeLa cells, which are p53-deficient.

**Figure 3:**
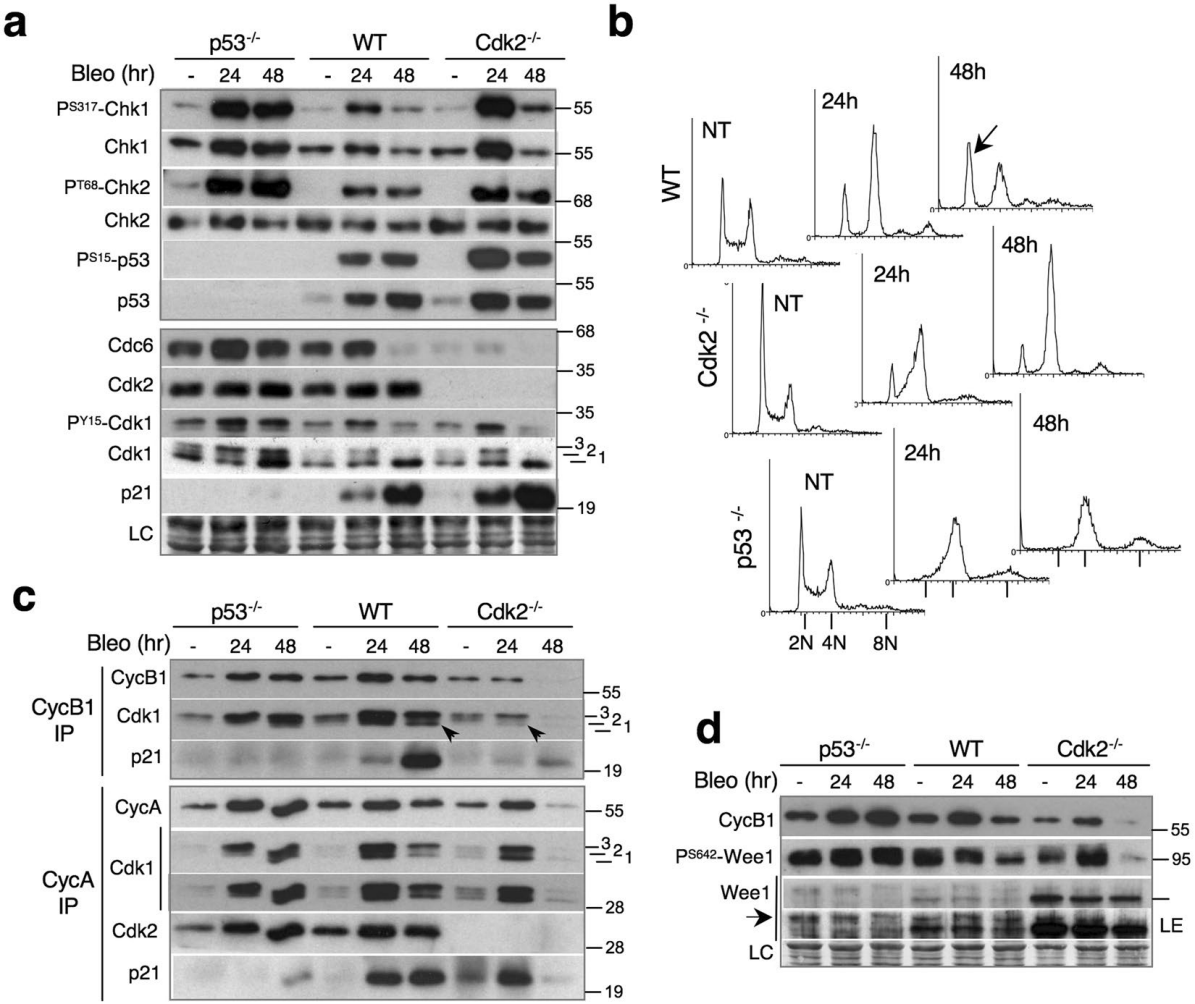
Cdk2 is not required for Chk1 activation upon DNA damage by bleomycin, and its absence slows S/G2 progression. (**a**) Immunoblots showing DNA damage response in WT, Cdk2^−/−^ and p53^−/−^ HCT-116 cells exposed to bleomycin for 24 h and 48 h. NT, non-treated cells. (**b**) FACS analysis of non-treated (NT) and WT, Cdk2^−/−^ and p53^−/−^ HCT-116 cells exposed to bleomycin 24 h and 48 h. Arrow shows a G1 population that escaped from G2 arrest. (**c**) Immunoblot analysis of CycA and CycB1 immunoprecipitates from WT, Cdk2^−/−^ and p53^−/−^ HCT-116 cells exposed to bleomycin. 1, 2 and 3 indicate different Cdk1 phospho-isoforms. (**d**) Immunoblots showing CycB1 and Wee1 phosphorylation (P^S642^) in non-treated (−) and WT, Cdk2^−/−^ and p53^−/−^ HCT-116 cells exposed to bleomycin for 24 h and 48 h. Arrow indicates slow SDS-PAGE mobility band that co-migrates with hyper-phosphorylated Wee1 (P^S642^-Wee1) band. LE, longer ECL exposure to appreciate SDS-PAGE shift of different Wee1 phospho-isoforms. Bar on the right indicates migration of 100 kDa molecular mass marker. LC, loading control. Uncropped images of blots are shown in Supplementary Fig. S17.

In contrast to HU (Fig. 1a), bleomycin induced strong phosphorylation of Chk1, Chk2 and p53, as well as an accelerated p21 induction in Cdk2^−/−^ HCT-116 cells (Fig. 3a). Furthermore, Cdk2 knockdown (KD) did not impair Chk1 and Chk2 phosphorylation in U2OS and HeLa cells (Supplementary Fig. S6). Thus, while Cdk2 and high Cdc6 levels might be important for ATR-Chk1 activation by HU, they are clearly dispensable for activation of ATR or ATM pathways by bleomycin. On the contrary, Cdk2 ablation strongly delayed S-G2 progression in bleomycin-treated cells. While a population of WT cells eventually progressed into mitosis (cf. increased G1 peak at 48 h), Cdk2^−/−^ cells remained stably arrested in G2 (Fig. 3b). This arrest was efficiently abrogated by caffeine, leading to cell cycle exit in G1 (Supplementary Fig. S7). Cdk2 depletion also delayed S-G2/M progression in bleomycin-treated U2OS and HeLa cells, as shown by FACS analysis and video-microscopy (Supplementary Fig. S6).

To determine the mechanisms that cause DNA damage-induced S/G2 delay in cells lacking Cdk2, we analyzed cyclin-CDK complexes that drive the G2/M transition. The most striking observation was that, whereas in both WT and p53^−/−^ cells, bleomycin induced strong accumulation of both CycB1-Cdk1 and CycA-Cdk1, in Cdk2^−/−^ cells arrested in G2, CycB1-Cdk1 complexes failed to increase (Fig. 3c, 24 h). In contrast, CycA-Cdk1 complexes accumulated normally, showing that this event is not dependent on Cdk2 and is uncoupled from increase of CycB1-Cdk1. Moreover, unlike in WT cells, where CycB1 associated with T14, Y15-dephosphorylated, active Cdk1 (arrows, isoform 1), in Cdk2^−/−^ and p53^−/−^ cells CycB1 was mainly bound to the hyper-phosphorylated Cdk1 isoform (3), which is inactive (Fig. 3c, arrow, see also Fig. 3a and model in Supplementary Fig. S8 for detailed explanation). Attenuated Cdk1 phosphorylation in these cells might be due to Chk1-dependent inhibition of Cdc25 family phosphatases, which dephosphorylate and activate Cdk1. Accordingly, at 24 h, Chk1 was more highly expressed and phosphorylated in Cdk2^−/−^ and p53^−/−^ cells than in WT cells (Fig. 3a). In addition, Chk1 also phosphorylates and activates Wee1 kinase^24^ that inhibits Cdk2 (Supplementary Fig. S8), which further stabilizes G2 arrest (Fig. 3d). Interestingly, Wee1 is over-expressed in Cdk2^−/−^ cells, which might facilitate rapid inhibitory phosphorylation of Cdk1 (Fig. 3d). However, Wee1 phosphorylation on Ser642 (PS642-Wee1) was much more elevated in p53^*−/−*^ cells where it associated with slower-migrating Wee1 species and correlated with stronger Chk1 activation (Fig. 3d, *arrow*). Thus, there is no evidence that Wee1 over-expression in Cdk2^*−/−*^ cells contributes to stronger G2 arrest.

In response to bleomycin, p21 increasingly associated with CycA in both WT and in Cdk2^−/−^ cells (Fig. 3c, 24 h) showing that Cdk1 replaced Cdk2 as a p21 target. This association, however, is not essential for G2 arrest since p53^−/−^ cells, that do not express p21 (Fig. 3a), also efficiently arrest in G2 (Fig. 3b). In these cells, stable G2 arrest is most likely due to strong and persistent Chk1 activation that stabilizes inhibitory Cdk1 phosphorylation on Y15 by Wee1 (Fig. 3a). As expected, in comparison to CycA, CycB1 was initially a poor target of p21 (Fig. 3c, 24 h). However, p21-bound CycB1-Cdk1 complexes markedly accumulated after 48 h (Fig. 3c), which is consistent with a role of p21 in their degradation associated with the cell cycle exit, rather than initial G2 arrest^25^. These complexes were virtually absent in Cdk2^−/−^ cells, presumably due to a strong down-regulation of cyclin B1 that occurred in parallel with induction of p21, and diminished phosphorylation of Chk1, Wee1 and Cdk1 (Fig. 3a,d, 48 h). In contrast, none of these events took place in p53^−/−^ cells (Fig. 3c), which stably arrest in G2 (Fig. 3b; see below).

We considered the possibility that delayed S-G2 progression in Cdk2^−/−^ cells might be due to Chk1 hyperactivation that, in turn, might prevent CycB1-Cdk1 increase (Fig. 3a). However, Chk1 is also strongly activated in p53^−/−^ cells (Fig. 3a), yet CycB1-Cdk1 levels increased to the same extent as in WT cells (Fig. 3c). Therefore, upregulated Chk1 alone cannot account for impaired CycB1-Cdk1 accumulation in Cdk2^−/−^ cells.

In summary, while Cdk2 is not required for Chk1 activation and G2 arrest by bleomycin, its inactivation (or absence) might restrain CycB1 increase and S-G2 progression, thereby facilitating onset of the G2 cell cycle exit program.

### Cdk2 downregulation accelerates DNA damage-induced cell cycle exit by reducing Cdk6

**Figure 4:**
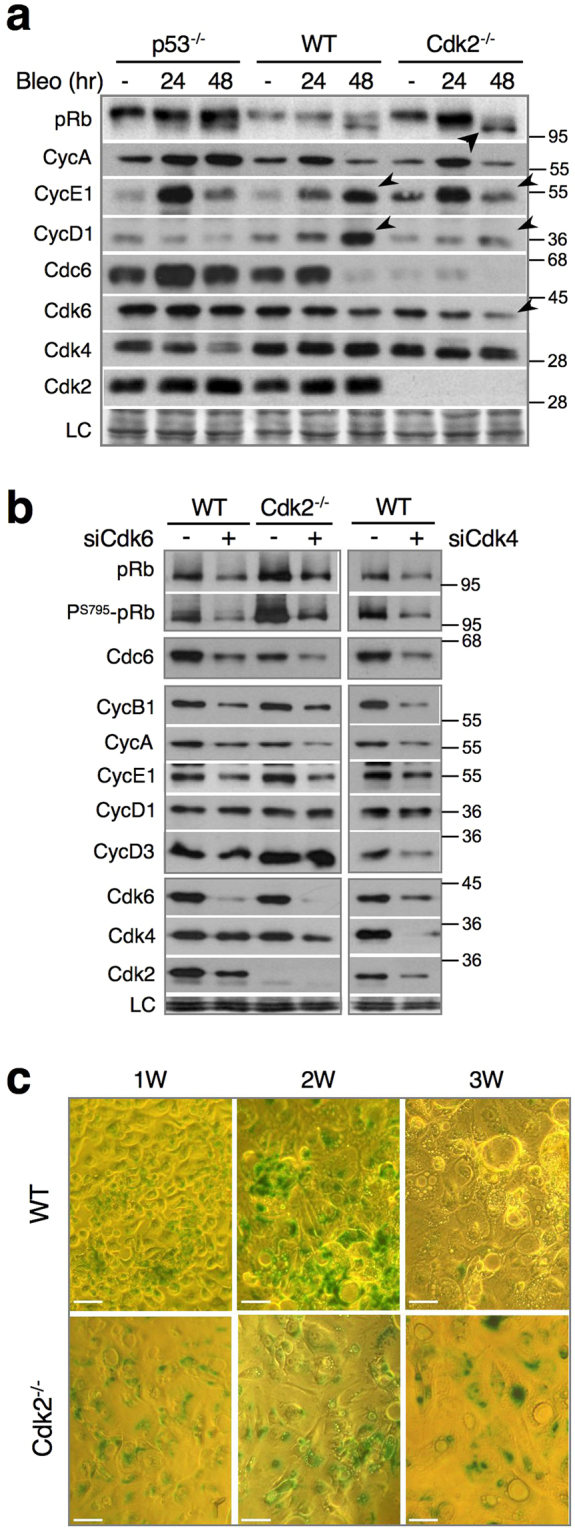
Cdk2 downregulation accelerates DNA damage-induced cell cycle exit by reducing Cdk6. (**a**) Immunoblot analysis showing indicated cell cycle regulators in wild-type (WT), Cdk2^−/−^ and p53^−/−^ HCT-116 cells exposed to bleomycin. Arrow indicates hypo-phosphorylated pRb accumulation. Arrowheads show increase of CycE1 and CycD1 after 48 h in WT cells. LC, loading control. (**b**) Immunoblot analysis of indicated cell cycle regulators in proliferating wild-type (WT) and Cdk2^−/−^ HCT-116 cells that were previously depleted for Cdk6 (siCdk6, left panel) or Cdk4 (only WT, right panel). Cells were harvested and lysed 48 h after transfection with Ctl-, Cdk6- or Cdk4-specific siRNA. LC, loading control. (**c**) β-galactosidase staining of WT and Cdk2^−/−^ cells 1 week (W), 2 weeks and 3 weeks after 24 h treatment with bleomycin. Bar, 10 μM. Uncropped images of blots are shown in Supplementary Fig. S17.

We observed that Cdk2^−/−^ cells arrested in G2 showed accelerated degradation of mitotic Cyclin-Cdk1 complexes (Fig. 3c,d), concomitant with diminished phosphorylation of Chk1 (Fig. 3a) and Wee1 (Fig. 3d). Together with p21-dependent inhibition of pRb and p130 phosphorylation and degradation of Cdc6 (Fig. 3a), these are hallmarks of cell cycle exit in G2, preceding senescence^23,25^. These changes were absent in p53^−/−^ cells, which lack p21 (Fig. 3c, Fig. 4a). Consequently, in p53^−/−^ cells, pRb remained hyper-phosphorylated even after prolonged DNA damage (Fig. 4a), indicating that, after G2 arrest, p53^−/−^ cells have not exited the cell cycle. Moreover, in Cdk2^−/−^ cells, pRb phosphorylation diminished faster than in WT cells. The same was true for expression of Cdk6, a D-type cyclin-associated kinase that phosphorylates pRb. Neither pRb phosphorylation nor Cdk6 expression was affected in p53^−/−^ cells (Fig. 4a, 48 h). Cdk2 depletion also strongly reduced Cdk6 levels in HeLa and U2OS cells (Supplementary Fig. S9). Thus, Cdk2 might delay the cell cycle exit by stimulating Cdk6 expression (see below).

To test functional significance of Cdk6 down-regulation, we depleted this pRb kinase in WT and Cdk2^*−/−*^ HCT-116 cells. Paradoxically, pRb phosphorylation was significantly and reproducibly increased in Cdk2^*−/−*^ cells (Fig. 4a,b). This is probably due to increased CycD3 and CycE1 levels (Fig. 4b). Indeed, in both WT and Cdk2^*−/−*^ HCT-116 cells, Cdk6 knockdown strongly inhibited pRb phosphorylation and led to marked downregulation of Cdc6, CycB1, CycA and CycE1 but it did not affect D-type cyclins (Fig. 4b, *left panel*). Notably, Cdk6 KD also reduced Cdk2 levels suggesting a feedback loop between these CDKs. These results indicate that Cdk6 might be crucial for pRb phosphorylation and maintenance of cell cycling even in the presence of Cdk4. Cdk4 knockdown had similar effect on pRb phosphorylation and on CycB1, CycA and CycE1 levels and, additionally, reduced Cdk6 and CycD3 levels (Fig. 4b, *right panel*). Taken together, these results suggest that upon DNA damage, Cdk2 downregulation/inactivation facilitates cell cycle exit, at least in part by decreasing Cdk6.

Finally, we wanted to test whether the accelerated cell cycle exit resulted in senescence. β-galactosidase (β-gal) staining indeed confirmed that cells exposed to bleomycin became senescent, but as it was only apparent by 1 week, it was not sensitive enough to corroborate faster entry of Cdk2^−/−^ cells in senescence (Fig. 4c). However, β-gal staining is more durable in comparison to WT cells, suggesting that Cdk2 loss promotes maintenance of the senescent phenotype (Fig. 4c, *48* *h*).

### CycE1-Cdk2, and not CycE1-Cdk1, is preferential p21 target in senescence

A characteristic feature of replicative senescence is accumulation of p21-bound and inactive CycE1-Cdk2 and CycD1-Cdk2 complexes^26,27^. More recently, concomitant accumulation of p21 and CycD1 was linked to irreversible cell cycle exit^28^. Indeed, exposure to bleomycin resulted in continuous increase of CycD1 and CycE1 in WT cells, whereas this was not the case in Cdk2^−/−^ or in p53^−/−^ cells (Fig. 4a, *48* *h*, *arrowheads*). These results suggest that the increase of G1 cyclins after DNA damage-induced cell cycle arrest/exit might be Cdk2-dependent.

**Figure 5:**
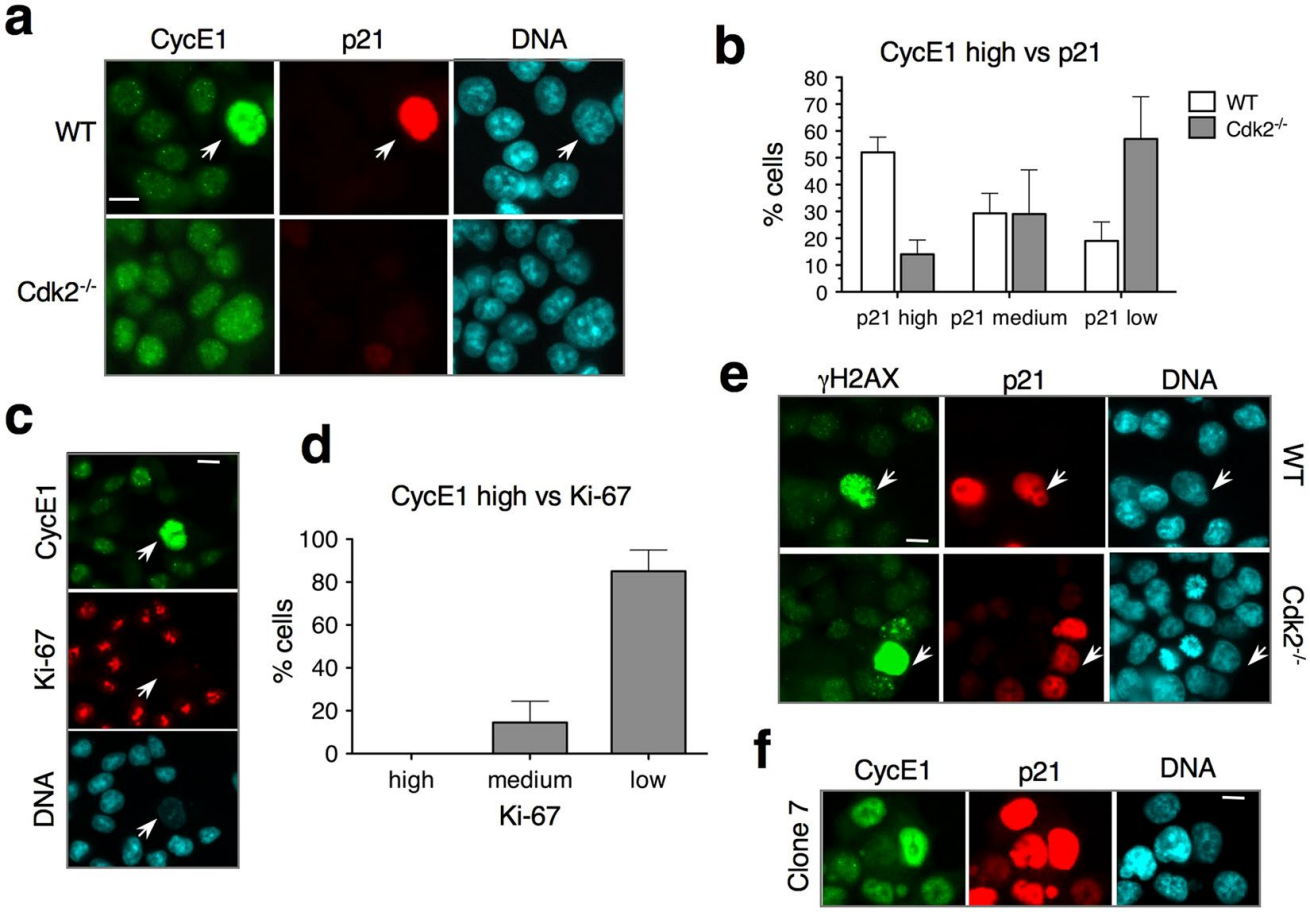
CycE1-Cdk2, and not CycE1-Cdk1, is preferential p21 target in senescence. (**a**) Immunofluorescence showing localization of CycE1 and p21 in proliferating WT and Cdk2^−/−^ HCT-116 cells. Arrow shows the cell with elevated levels of both CycE1 and p21. Bar, 10 μM. See Supplementary Fig. S10 for larger fields. (**b**) Cells expressing high CycE1 levels were scored for p21 expression. Mean and standard deviation of three separate experiments are given. For more detailed explanation of the criteria used for quantification see Materials & Methods. (**c**) Immunofluorescence showing co-localization of CycE1 and Ki-67 in proliferating WT HCT-116 cells. Arrow shows the cell with elevated levels of CycE1 with low Ki-67 signal. Bar, 10 μM. See Supplementary Fig. S12 for larger fields. **(d)** Cells expressing high CycE1 levels were scored for Ki-67 expression. Mean and standard deviation of three separate experiments are given. For more detailed explanation of the criteria used for quantification see Materials & Methods. (**e**) Immunofluorescence showing proliferating HCT-116 cultures with subpopulation of cells with strong γH2AX and p21 signals. Bar, 10 μM. See Supplementary Fig. S13 for larger fields.** (f) **Immunofluorescence showing proliferating cultures of Cdk2^−/−^ HCT-116 cells with ectopic expression of Cdk2 (clone 7) that restored accumulation of p21 and CycE1. Bar, 10 mM.

In line with this idea, we found that asynchronously growing WT HCT-116 cultures contained a significant cell population (3–6% cells, n > 600) expressing abnormally elevated CycE1 levels. These cells invariably expressed high levels of p21 (Fig. 5a,b and Supplementary Fig. S10), lacked CycA (*see* Supplementary Fig. S11) and displayed a weak Ki-67 signal (Fig. 5c,d and Supplementary Fig. S12), which is another hallmark of senescence^29^. The origin of these noncycling cells could be DNA damage lesions due to deregulated DNA replication, which is often present in cancer cells^30^. Indeed, cells with strong γH2AX signal or upregulated p21 were frequently observed in both WT and Cdk2^−/−^ HCT-116 cultures (Fig. 5e and Supplementary Fig. S13). In contrast, very few CycE1-overexpressing cells with elevated p21 levels were observed in Cdk2^−/−^ HCT-116 cultures (Fig. 5a,b and Supplementary Fig. S10). Importantly, ectopic expression of Cdk2 restored accumulation of these cells (Fig. 5f). Thus, although in HCT-116 cells, Cdk1 replaced Cdk2 in most p21-bound cyclin-Cdk complexes (Fig. 3c), this is apparently not the case in senescence, where CycE1 (and CycD1) accumulate (Fig. 4a). These data suggest that, during senescence onset in HCT-116 cells, p21 specifically targets and stabilizes CycE1-Cdk2 (and probably, CycD1-Cdk2) complexes. Cells lacking Cdk2 would thus more readily exit the cell cycle.

### Cdk2 KD strengthens the G2/M checkpoint and promotes DNA damage-induced cell cycle exit in U2OS cells by reducing Cdk6

**Figure 6:**
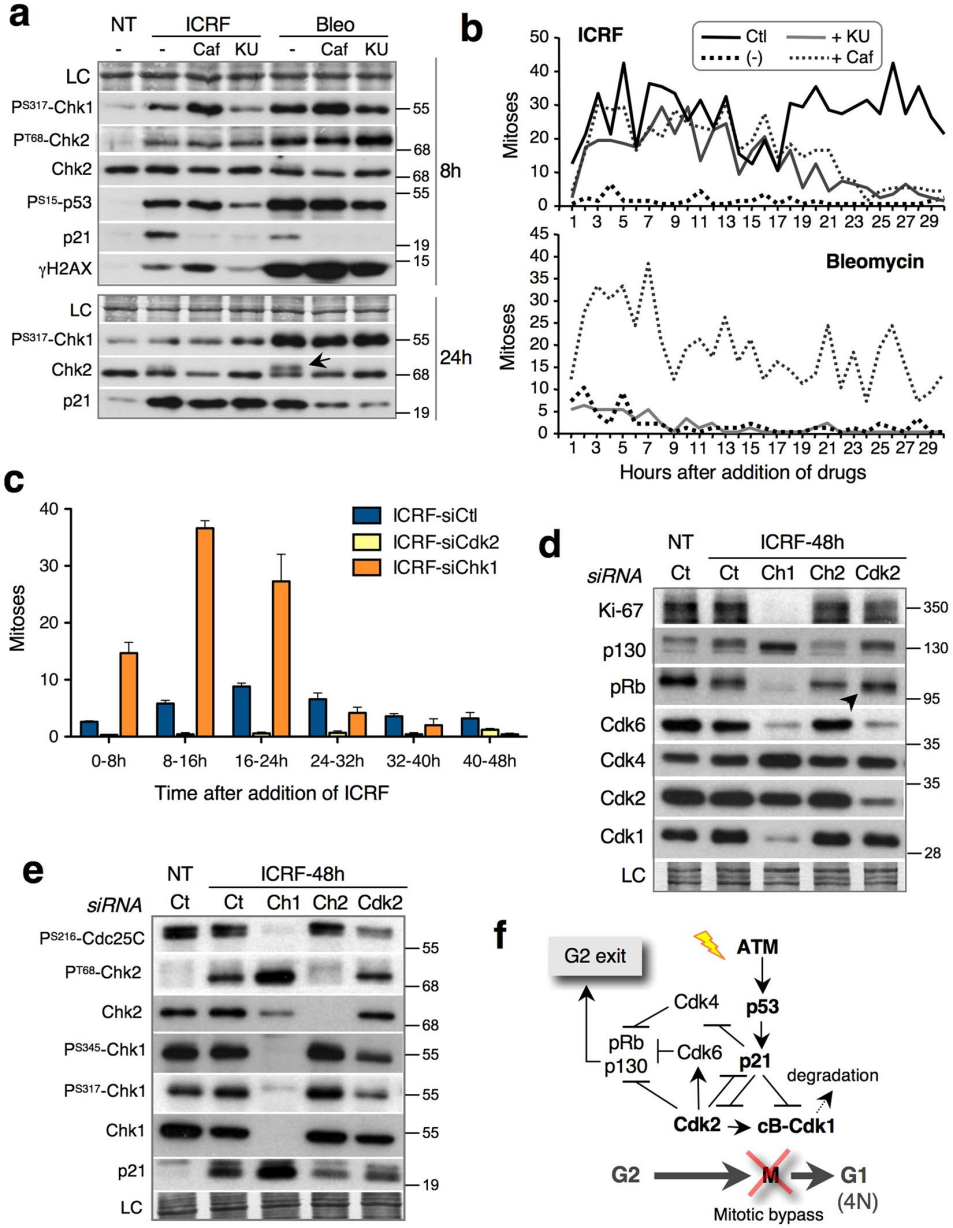
Cdk2 KD strengthens the G2/M checkpoint and promotes DNA damage-induced cell cycle exit in U2OS cells by reducing Cdk6. (**a**) Immunoblots showing effects of KU-55933 (KU) and caffeine (Caf) on Chk1 phosphorylation in ICRF- and bleomycin-treated U2OS cells. Arrow, ATM-mediated Chk2 mobility shift; NT- non-treated cells. (**b**) Occurrence of mitoses based on video-microscopy data of non-treated (Ctl) and U2OS cells exposed to ICRF (top) and bleomycin (below) in the absence (−) or presence of Caffeine (+Caf) or KU-55933 (+KU). Mitoses were counted at 2-hour intervals for 30 hours and normalized to the total cell number, as described in Methods. (**c**) Video-microscopy data showing mitoses in ICRF-treated control (siCtl), Cdk2 KD (siCdk2) and Chk1 KD (siChk1) U2OS cells. Mitoses were counted at 8-hour intervals. Mean and standard deviation of three separate experiments are given. See also Supplementary Fig. S15. (**d**) Immunoblots showing effects of Control (Ct), Chk1 (Ch1), Chk2 (Ch2) and Cdk2 depletion on pRb pathway in ICRF-treated U2OS cells. Arrow shows hypo-phosphorylated pRb. LC, loading control. (**e**) Immunoblots showing effects of Control (Ct), Chk1 (Ch1), Chk2 (Ch2) and Cdk2 depletion on DNA damage response regulators in ICRF-treated U2OS cells. LC, loading control. (**f**) Model: How Cdk2 inhibition promotes implementation of G2 exit program after DNA damage. See Supplementary Fig. S16 for more details. Uncropped images of blots are shown in Supplementary Fig. S17.

Our results showed that in cells exposed to DNA damage, Cdk2 knockout slows down S-M progression and accelerates cell cycle exit. We reasoned that the S/G2 delay in Cdk2^−/−^ HCT-116 cells could be due to the stronger Chk1 activation provoked by bleomycin (Fig. 3a). Therefore, we wished to test whether Cdk2 depletion could reinforce the G2 arrest in the presence of ICRF, which generates weaker DNA damage response (Fig. 6a) resulting in less efficient G2/M checkpoint that can be over-ridden. However, as shown by video-microscopy, ICRF does not induce G2 arrest in either WT or Cdk2^−/−^ HCT-116 cells; instead, the cells arrest after mitosis with aberrant nuclei (Supplementary Fig. S14). This phenotype is presumably due to insufficient activation of ATM/ATR-Chk1 pathway by ICRF in this cell line^23^, a defect that could not be compensated (rescued) by Cdk2 knockout.

We therefore studied U2OS cells, in which ICRF more efficiently blocks G2/M progression^23^, due to stronger Chk1 activation (Fig. 6a). The G2 arrest by ICRF could be readily abrogated by the ATM inhibitor KU-55933 (KU), which inhibits activation of p53, Chk1 and Chk2 and impairs p21 induction (Fig. 6a,b). In contrast, in bleomycin-treated cells, despite KU-55933 strongly inhibiting p21 induction and Chk2 activation, Chk1 activity remained sufficient to maintain G2 arrest. Caffeine, which bypasses both ATR and ATM pathways^31^, promoted mitosis in both bleomycin and ICRF-arrested U2OS cells (Fig. 6b).

As expected, U2OS cells exposed to ICRF arrested efficiently in G2, with only a minor cell population progressing to mitosis (siCtl, Fig. 6c and see Supplementary Fig. S15). In agreement with our hypothesis, Cdk2 KD completely abolished this “mitotic leak” and re-enforced G2 arrest (Fig. 6c). Conversely, Chk1 KD, and to a lesser extent Chk2 KD, abrogated G2 arrest by ICRF (Fig. 6c and Supplementary Fig. S15). Next, we asked whether Cdk2 depletion promoted markers of cell cycle exit in G2-arrested cells. Indeed, Cdk2 KD caused a significant decrease of Ki-67 and accumulation of hypo-phosphorylated p130 and pRb after 48 h of ICRF treatment. In comparison, Chk1 KD, which we have recently found to accelerate DNA damage-induced cell cycle exit (Lossaint *et al*., submitted), caused an even stronger change in these markers (Fig. 6d), probably by stimulating p21 accumulation (Fig. 6e). Notably, like Chk1 KD, Cdk2 KD strongly reduced levels of pRb kinase Cdk6 (Fig. 6d), which corroborates the results shown in Fig. 4a and in Supplementary Figs S1 and S9 and underscores the role of Cdk6 for maintaining the pRb phosphorylation and cell cycle progression (Fig. 4b). Finally, Cdk2 depletion markedly reduced phosphorylation of Chk1 and Cdc25C (Fig. 6e), which is consistent with promotion of the cell cycle exit programme. Taken together, these results demonstrate that suppression of Cdk2 strengthens the G2/M checkpoint, even in the absence of strong Chk1 activation. Moreover, they underline the involvement of Cdk2 inhibition in Cdk6 downregulation and the onset of cell cycle exit in G2 (Fig. 6f).

## Discussion

Here, we addressed the role of Cdk2 in the intra-S checkpoint and in DNA damage-induced G2 arrest in both p53-proficient and deficient cancer cell lines. Several reports implicated Cdk2 as a positive regulator of the DNA damage response^11–15,32^. These results were ostensibly at variance with data showing that CycA-Cdk2 complexes are major p21 targets in G2 arrest both in non-transformed^17^ and in transformed human cells^16^, which implied that Cdk2 inhibition is an integral component of the G2/M checkpoint. In this study we show that while Cdk2 plays a positive role in the G1/S arrest by replication stress, its absence rather promotes onset of the G2 cell cycle exit program in response to DNA damage due to accelerated p21 induction, reduced accumulation of CycB1-Cdk1 and down-regulation of pRb kinase Cdk6 (Fig. 6f).

One of the mechanisms whereby Cdk2 is proposed to regulate the DNA damage response is activation of the ATR-Chk1 pathway^12–14^, possibly *via* phosphorylation of Cdc6 and ATRIP^13^. Additionally, Cdk2 was shown to phosphorylate the MRN complex protein Nbs1^15^, which is involved in resection of double-strand breaks and leads to ATM/ATR signalling^33^. Our results showing that Chk1 phosphorylation and G1/S arrest by hydroxyurea (HU) are strongly impaired in Cdk2^−/−^ HCT-116 cells are consistent with these data (Supplementary Fig. S16). The replication stress response depends on the level of DNA replication and of nucleotide pools, both of which are controlled by Cdk2^34^. It was recently found that inhibiting Wee1, the negative regulator of Cdk1/2, caused loss of ribonucleotide reductase subunit 2 (RRM2) due to upregulation of CDK-mediated degradation. Conversely, Cdk2 inhibition restored RRM2 levels in cancer cells with low expression of this enzyme^35^. Thus, although Cdk2 ablation did not affect RRM2 levels in HCT-116 cells, the role of Cdk2 in hydroxyurea responses might rather be indirect, perhaps by controlling the magnitude of the replication stress through control of numbers of replication forks. Nevertheless, while Cdk2 knockdown (KD) in p53-proficient U2OS and HCT-116 or p53-deficient HeLa cancer cell lines markedly diminished Chk1 phosphorylation by HU, the effect on S-phase arrest was rather modest. Our data imply that residual Cdk2 could be responsible for the absence of more pronounced G1/S arrest phenotype in these Cdk2 KD cells, suggesting a threshold effect. Cdk2 was found to have a more pronounced effect on cell cycle entry in low serum conditions^7^. It would be interesting, and possibly of clinical relevance, to see if different growth conditions can also change the dependency of individual cancer cell lines on Cdk2 for their arrest in response to replication poisons.

In contrast to the S-phase checkpoint, we found no evidence that Cdk2 and normal Cdc6 levels are required for Chk1 phosphorylation or G2 arrest onset by sustained DNA damage (bleomycin) in any of the cell lines studied, including HeLa cells in which p21 is not induced. On the contrary, whereas Cdk2 knockout/knockdown did not affect cell division in these cell lines, the lack of Cdk2 markedly impaired S-G2-M progression in the presence of DNA damage. In Cdk2^−/−^ HCT-116 cells, unlike CycA-Cdk1, CycB1-Cdk1 complexes failed to accumulate in arrested cells. This result corroborates previous findings showing that CycA-Cdk2 activates CycB1-Cdk1 by positively regulating Cdc25 phosphatases^36^ and inhibiting Wee1^37^. Moreover, this underlines the importance of inhibition of CycA-Cdk2 by p21 for efficient G2/M checkpoint. Finally, our data are consistent with a previous study in mice showing that overall levels of CDK activity are important for DNA damage responses, rather than any particular CDK^10^. Based on these results we propose that, upon DNA damage, Cdk2 inhibition slows S-M progression to allow implementation of a G2 exit program (see below, Fig. 6f and Supplementary Fig. 16).

How can this model be reconciled with the data showing that, in the absence of p53, Cdk2 is required for Chk1 activation and G2 arrest^13^? It was proposed that the lack of the G2 arrest phenotype in Cdk2^−/−^ HCT-116 cells is masked by the action of the p53-p21 pathway, which inhibits Cdk1^13^. While the present and previous studies demonstrate that in several frequently-studied p53-proficient cancer cell lines (HCT-116, U2OS, MCF7) CycB1-Cdk1 complexes are minor p21 targets^16,21,23^, we cannot exclude that p21 contributes to G2 arrest by inhibiting Cdk1 associated with CycA. It is thus possible that, in the absence of p21 and persistent Chk1 activity, Cdc25A upregulation caused by Cdk2 depletion eventually results in entry into mitosis despite initial efficient G2 arrest^13^. However, based on our results that support a role of Cdk2 inhibition in G2 arrest, we conclude that Cdk2 is not required for an efficient DNA damage response in all circumstances, but, rather, timely inactivation of Cdk2 might be necessary. At first sight, our conclusion appears to be contradictory to a study using analogue-sensitive Cdk2 knockin cells, where specific Cdk2 inhibition was found to sensitise cells to irradiation^15^. This was interpreted as being due to a defective G2/M checkpoint. However, we propose that the latter finding might rather be due to a role of Cdk2 in preventing exit from the cell cycle upon irradiation. Either a lack of G2/M checkpoint and consequent mitotic catastrophe, or increased cell cycle exit, would give the same results: loss of colony formation.

Despite its predominant association with CycA and Cdk2^16,17^, the significance of inactivation of CycA-Cdk2 by p21 upon G2 arrest was unclear. Although it had been proposed that p21 blocks phosphorylation of the pRb family by inhibiting CycA-Cdk2 to induce cell cycle exit in G2^17,38^, there had so far been little evidence confirming this hypothesis. Our current results reveal that the absence of Cdk2 accelerates DNA damage-induced cell cycle exit program in p53/pRb-proficient cells, possibly by enhanced p21 induction and through inhibition of Cdk6 expression (Fig. 6f and Supplementary Fig. S16). Notably, while confirming previous findings that Cdk2 is dispensable for inactivating phosphorylation of pRb family proteins^1,7^, owing to association of CycE1 and CycA with Cdk1 and upregulation of CycD3 and CycE1, we showed that Cdk2 depletion led to strong and specific down-regulation of Cdk6, especially after DNA damage. Although Cdk6 is widely considered as a redundant Cdk4 homolog, pRb phosphorylation is lower in Cdk6^−/−^ than in Cdk4^−/−^ or Cdk2^−/−^ MEFs^1^. Consistent with this observation, we found that siRNA-mediated Cdk6 depletion strongly blocked pRb phosphorylation even in the presence of Cdk4, which resulted in marked down-regulation of Cdc6, CycB1 and CycE1. This indicates that, at least in HCT-116 cells, Cdk1 cannot replace Cdk6 in D-type cyclin complexes. Therefore, while under unperturbed conditions Cdk1 and Cdk4 may compensate for the absence of Cdk2 and low Cdk6 levels (upon Cdk2 KD), this does not seem to be sufficient in the presence of DNA damage. In this case, Cdk1 and Cdk4 are inhibited by p21, which together with Cdk6 downregulation, blocks phosphorylation of pRb and p130 and induces cell cycle exit in G2 (Fig. 6f). The importance of Cdk6 is further underlined by the recent results showing that Cdk6 amplification is paramount for the resistance of cancer cells to CDK4/6 inhibitors^39^. In addition to its role as pRb/p130 kinase, Cdk6 was shown to be a transcriptional regulator implicated in tumorigenesis^40^. Thus, by targeting CycA-Cdk2, p21 might drive G2 exit by slowing down S-G2 progression and blocking pocket protein phosphorylation, either directly or by repressing Cdk6 (Fig. 6f). Unravelling this response will require future studies.

Our work also highlights the importance of CycE1-Cdk2 complexes as p21 targets during the DNA damage-induced cell cycle exit. Paradoxically, although overexpression of G1 cyclins is usually associated with cancer^41^, inactive CycE1-Cdk2 and/or CycD1-Cdk2 complexes were shown to accumulate during cellular senescence^26,27,42^. More recently, a strong increase of CycD1 was also observed upon DNA damage-induced G2 arrest^43^ and co-accumulation of CycD1 and p21 was functionally associated with the permanent cell cycle exit program^28^. The stabilization of G1 cyclin-CDK complexes is in striking contrast with a rapid degradation of mitotic cyclin-CDK complexes preceding the cell cycle exit^25^. What could be a function of G1 cyclins in (permanently) arrested cells? It has been suggested that CycD1 (when over-expressed?) might have a role in blocking proliferation^28,42^ or in DNA repair and stability^44,45^. However, one could only speculate about the role of p21-bound CycE1-Cdk2 complexes.

At first sight, the requirement for inhibition of Cdk2 to promote cell cycle exit might appear to be at odds with data showing that Cdk2 is dispensable both for cell cycle arrest and for tumour inhibition by CDK inhibitors^46^. This is probably because all Cdk2 partners can also bind to Cdk1 to ensure cell cycle progression^1,47^ (this study). On the other hand, our results are fully consistent with those showing that Cdk2 depletion promotes Myc-induced cellular senescence, which is characterized by the absence of p21 induction^18^. Although the role of CycA-Cdk2 inhibition was not addressed in recent work describing the G2 exit program^20,21,38^, more recent data showed that, *in cellulo*, a drop of Cdk2 activity preceded CycB1 degradation during G2 exit^48^. In conjunction with this observation, our results provide evidence for the critical role of p21-mediated Cdk2 inhibition in DNA damage-induced cell cycle exit in G2. We suggest that specific Cdk2 inhibitors should not be toxic to normal dividing cells but they might promote senescence of cancer cells when combined with DNA damaging agents. Therefore, in line with recent results describing the Cdk2 activity as a signature predicting cancer risk^49^, we propose that Cdk2 should be re-considered as a potential cancer therapeutic target^41^.

## Methods

### Cell lines and drug treatments

Wild-type, p53^−/−^ and p21^−/−^ human colon carcinoma cells HCT-116 were a generous gift of Dr. B. Vogelstein (Baltimore, USA). The parental and Cdk2^−/−^ HCT-116 cells were purchased from Horizon Discovery UK (HD R02-015, Cambridge, UK) and they were obtained according to the original protocol published previously^13^. The human osteosarcoma U2OS and HeLa cell lines were purchased from the ATCC. Excepting for HCT-116 cells (McCoy’s 5 A medium) all cell lines were cultured in Dulbecco modified Eagle medium (DMEM - high glucose, pyruvate, GlutaMAX – Gibco® Life Technologies) supplemented with 10% foetal bovine serum (SIGMA or HyClone). Cells were grown under standard conditions at 37 °C in a humidified incubator containing 5% CO_2_.

Radiomimetic agent bleomycin (10 μg/ml) and Topoisomerase II inhibitor ICRF-193 (bis(2,6-dioxopiperazin), 2 μg/ml) were added to asynchronously growing cells as described previously^17^. The ATM inhibitors, caffeine (Sigma-Aldrich, St Louis, MO, USA) and KU-55933 (Kudos Pharmaceuticals, Cambridge, UK), were added one hour before genotoxic agents, at concentrations of 5 mM and 10 μM, respectively. Ribonucleotide reductase inhibitor, hydroxyurea (2 mM), was added to asynchronously growing cells for 20 h.

### Flow cytometry - cell cycle analysis

Cells were harvested, washed once with cold PBS (+1% BSA), resuspended in 300 μl cold PBS and fixed with 700 μl chilled 100% ethanol, and stored at 4 °C. On the day of analysis, cells were pelleted by centrifugation at 5000 rpm (5 min). After washing once with 1% BSA in PBS, cells were stained with Propidium Iodide staining solution (10 µg/ml Propidium Iodide, 1% BSA, 200 µg/ml RNase A in PBS) for 30 min at room temperature and analysed with BD FACS Calibur (BD Biosciences, San Jose, CA).

### Cdk2-expressing clones

*CDK2* gene was cloned into pTracer™-CMV2 Mammalian Expression Vector (ThermoFisher Scientific) that expresses GFP fused to selectable marker Zeocin™. HCT116 Cdk2^−/−^ cells were transfected with the plasmid using Lipofectamine® 2000 according to the protocol (ThermoFisher Scientific) and treated with 800 μg/ml Zeocin™ during 10 days. Cells were further diluted to 0, 5 cells/well in 96-well plate in order to obtain individual clones. Clones were expanded and kept under selection prior to the experiments.

### Video-microscopy experiments

Conditions for video-microscopy experiments were described previously^23^. We used inverted wide-field microscope (Leica DMIRE2, objective Leica 10X HC PL FLUOTAR 0.3 PH1, Camera Micromax YHS 1300) equipped with CO_2_-, temperature- and humidity-controlled chamber (Montpellier RIO Imaging facility-MRI). Mitoses (rounded cells) were scored by inspection of video-microscopy sequences (Metamorph software). The images were taken at 10–15 min interval for at least 48 hours. Three fields for each situation were analyzed and normalized for the cell number at the beginning of the time-lapse sequence (% of cells at the start). Every experiment was repeated at least two times. For mitosis-entry kinetics, total number of mitotic cells during the given interval was plotted. For each experiment, all the conditions, including controls with untreated cells transfected with different siRNAs, were tested in parallel.

### Cell extracts, immunoprecipitation and immunoblots

Preparation of cell lysates, conditions for immunoprecipitation, p21 depletion experiments, histone H1 kinase assays and immunoblotting experiments have been described previously^17,50^. To detect P^S317^-Chk1, P^S345^-Chk1 and phosphorylation-induced mobility shifts of Chk2, pRb and p130, extracts were analyzed on 7.5% SDS-PAGE gels. Other cell cycle regulators and P^T68^-Chk2 were analyzed on 12.5% SDS-PAGE gels. For loading controls (LC) the scans of amido-black-stained PVDF membranes for each gel were shown.

Most of the primary antibodies were described in our earlier publications^17,19,23,51^. In addition, we have used: cyclin D2 (M-20, Santa Cruz Bio.), cyclin D3 (D-7 and B-10, Santa Cruz Bio), Cdk2 (D-12 and M-2; Santa Cruz Bio.), Cdk4 (C-22, Santa Cruz Bio.), Cdk6 (C-22 and B-10, Santa Cruz Bio.), Cdc6 (180.2, Santa Cruz Bio.), RRM2 (N-18, Santa Cruz Bio.), Cdc25C phospho-S216 (Cell Signalling), cyclin D1 (DCS-6, Cell Signalling), Ki67 (Abcam, ab16667), Rb phospho-S795 (Abcam, ab47474). Secondary antibodies for immunoblot analysis were anti-mouse (DAKO, Glostrup, Denmark) and anti-rabbit IgG HRP-conjugates (Promega, Madison, WI, USA) and protein AG coupled to HRP (Pierce, Rockford, IL, USA). The detection system used was Western Lightning Plus-ECL (PerkinElmer) and Amersham Hyperfilm^TM^ (GE Healthcare).

### Immunofluorescence

For the panels showing immunofluorescence images, a representative field was shown. Every experiment was performed at least two times with each genotoxic agent, and in several different cell lines. For each situation, at least ten images (40x magnification) were taken. Experimental conditions for immunofluorescence were described previously^17,19^. Immunofluorescence and digital interference contrast images were captured (microscope Leica CTR6000, objective Leica 40X HCX PL APO 1.25–0.75 oil, camera Coolsnap HQ2) and a composite generated using Adobe Photoshop (Adobe Systems Inc, San Jose, CA, USA) and Microsoft PowerPoint (Microsoft Corp, Redmond, WA, USA) softwares.

For the results presented in Fig. 4d,f immunofluorescence signal was quantified using ImageJ software. The DAPI images were used to identify the individual nuclei using a background correction, a mask creation based on threshold, a watershed segmentation and the analyse particles function in ImageJ. The total intensity of individual nuclei in p21 or Ki-67 and CycE1 images were quantified. 3 classes of object were created based on an arbitrary threshold: low, medium and high intensity. The % of cells per class was calculated for the each condition with the Excel Microsoft software. The population with high CycE1 intensity corresponded to 3–7% of cells.

### Beta-galactosidase experiments

HCT-116 cells were seeded on coverslips in 12-well plate, in a density of 100.000 cells per well. Cells were treated after 24 h with bleomycin (10 μg/ml). The drug was washed away from the cells after 48 h of incubation, followed by staining after 3 weeks using Senescence detection kit, according to the protocol (Abcam, ab655351). Photos were taken using an upright microscope magnification 20x.

### siRNA transfections

The SMARTpool ON-target (Cdk2, Cdk6, Cdk4, Chk1 and Chk2) were purchased from GE Dharmacon Research (Lafayette, CO, USA). As control, we used siRNA for luciferase, 5′- ACUGACGACUCUGCUACUC-3′ (Eurogentec, Seraing, Belgium) or ON-TARGETplus non-targeting controls siRNAs (D-001810-10) from DE Dharmacon.

Asynchronously growing cells were transfected with siRNA (100 nM) by a standard Ca-phosphate protocol. Cells were plated at density of 1.5 × 10^4^/cm^2^ the day before transfection. 30 minutes prior transfection, growing medium with antibiotics were exchanged for medium without antibiotics. Calcium phosphate–DNA co-precipitate was prepared (44 µL sterile H_2_O, 5 µL of 2.5 M CaCl_2_ and 1 µL of 100 μM siRNA (final concentration - 100 nM in medium), without vortexing. 50 µL CaCl_2_-siRNA solutions were combined with equal volume of 2xHBSS buffer (50 mM HEPES, 280 mM NaCl, 15 mM Na_2_HPO_4_, 10 mM KCl, mH 7.04). Co-precipitates were incubated at room temperature for 1 min, mixed by pipetting, added drop by drop into medium above cells and gently mixed. 24 hours after transfection, the cells were exposed (or not) to different genotoxic agents and harvested for biochemical or immunofluorescence analysis at indicated times or monitored by time-lapse microscopy. For some experiments (absence of DNA damage) the cells were harvested and analysed 48 hours after transfection.

### Data availability

The datasets generated during and/or analysed during the current study are available from the corresponding author on reasonable request.

## Electronic supplementary material

Supplementary Figures
