# Supplementary material for: Cdk2 strengthens the intra-S checkpoint and counteracts cell cycle exit induced by DNA damage
Source: Sci Rep. 2017 Oct 18;7:13429. doi: 10.1038/s41598-017-12868-5 (PMC5647392; doi:10.1038/s41598-017-12868-5)
Supplement: Supplementary file 1 — Supplementary Figures [file 41598_2017_12868_MOESM1_ESM.pdf]

Supplementary information

CDK2 Strengthens the intra-S checkpoint and counteracts cell cycle exit induced by DNA damage

Katarina Bacevic[1] , Gérald Lossaint[1,2], Thiziri Nait Achou[1], Virginie Georget[1], Daniel Fisher[1], and Vjekoslav Dulic[1,*]

1. IGMM, CNRS, Univ. Montpellier, Montpellier, France
2. Present address: Swiss Institute for Experimental Cancer Research (ISREC), Ecole Polytechnique Fédérale de Lausanne (EPFL), 1015 Lausanne, Switzerland

(*) Corresponding author: vjekoslav.dulic@igmm.cnrs

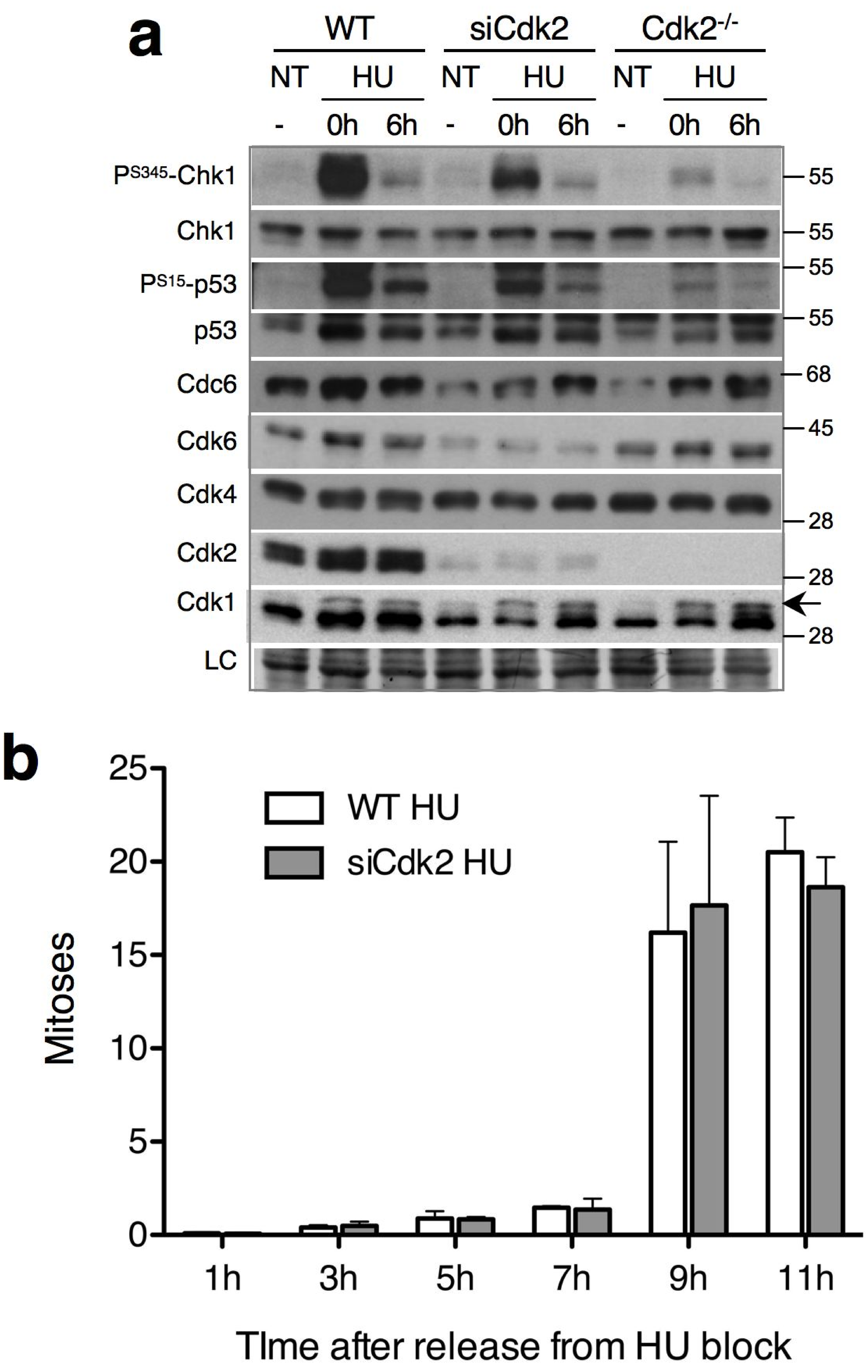

Supplementary Figure S1. Cdk2 is required for efficient Chk1 activation.

a. Immunoblots showing phosphorylation of Chk1 and p53 as well as the expression of indicated cell cycle regulators after 20h in hydroxyurea (HU-0h) and 6 hrs after release from the G1/S block in wild-type (WT), siCdk2 and $Cdk2^{-/-}$ HCT-116 cells. Arrow shows hyper-phosphorylated Cdk1. NT, non-treated cells. LC, loading control.

b. Video-microscopy data showing mitotic entry upon release from the HU block in wild-type (WT) and Cdk2 knockdown (KD) HCT-116 cells. Mean and mean deviation of four separate experiments are given.

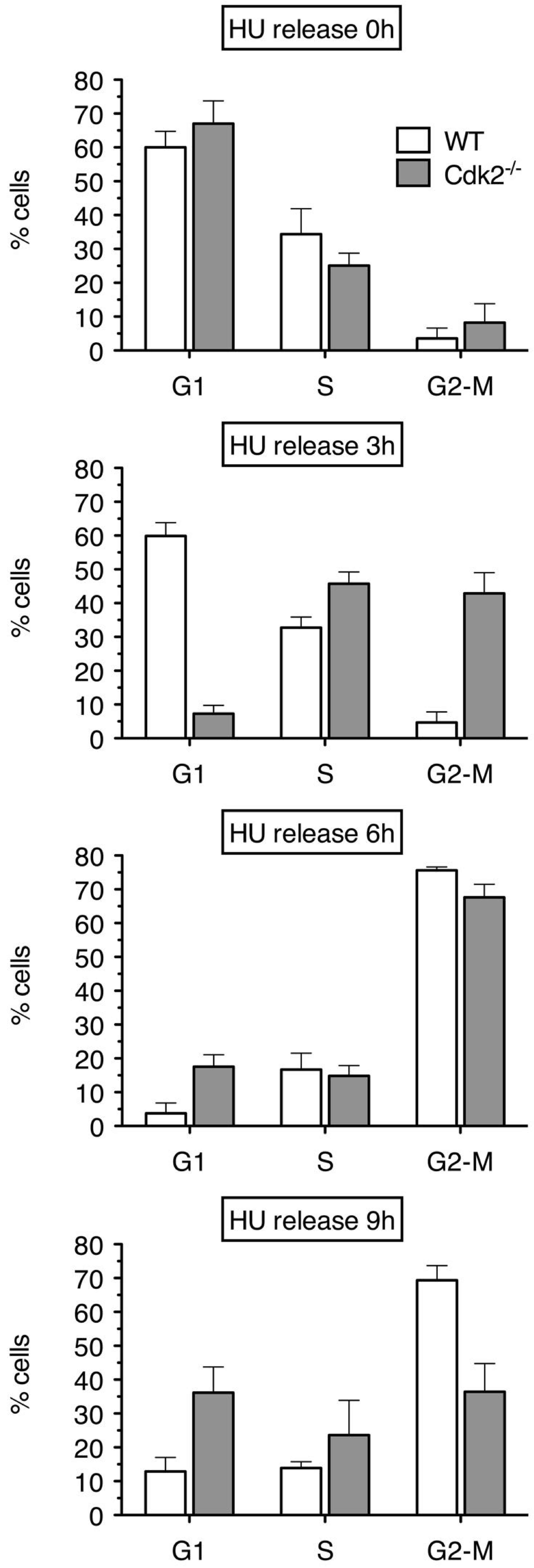

Supplementary Figure S2. Cdk2 is required for efficient intra-S checkpoint activation.
Quantification of flow cytometry (FACS) data from three independent experiments using FlowJoTM v.10.2 software (LLC 2006-2016) program, which shows cell cycle progression after release from the G1/S block by hydroxyurea (2mM) in wild-type (WT) and $Cdk2^{-/-}$ HCT-116 cells. Mean and mean deviation of three separate experiments are given.

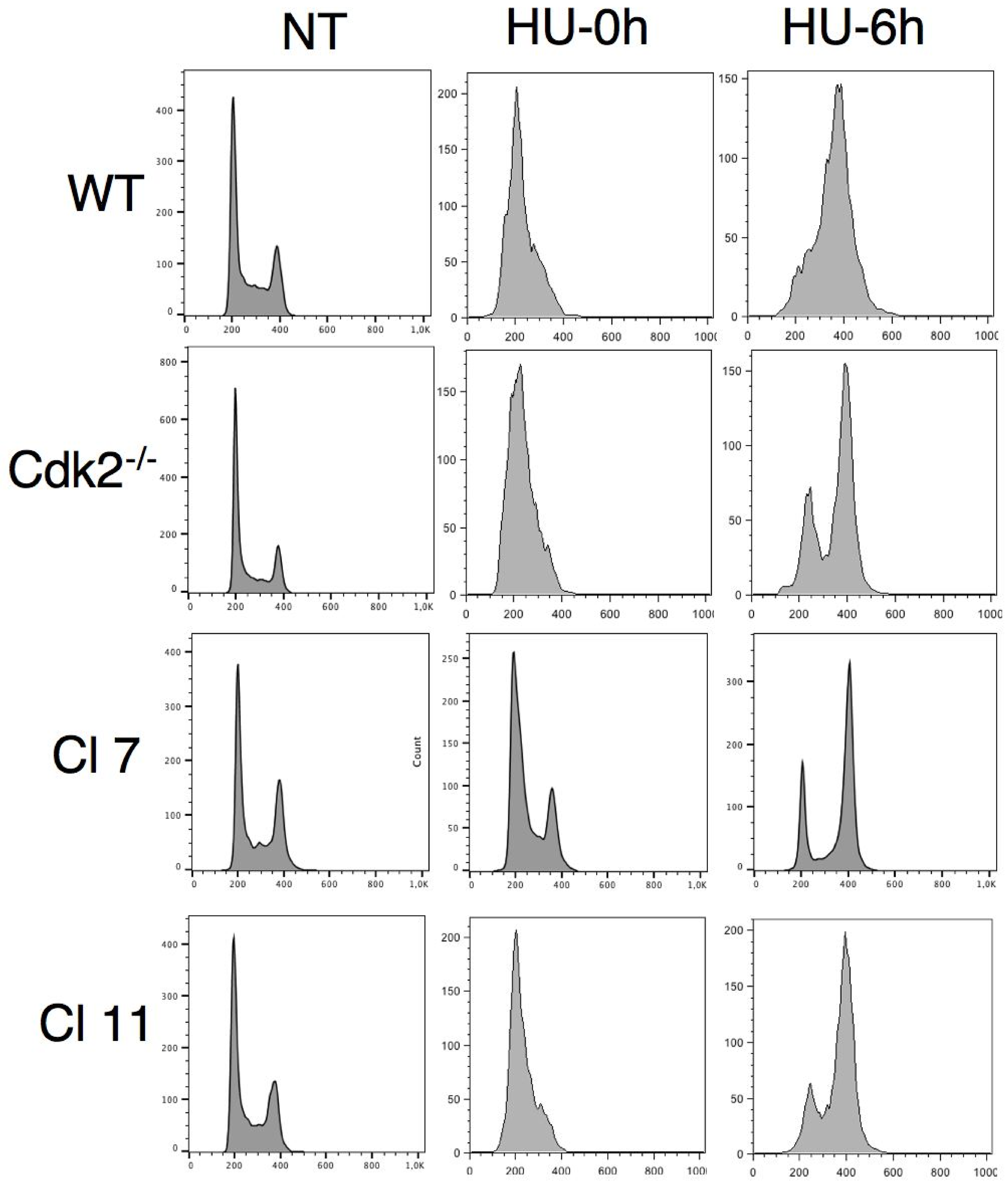

Supplementary Figure S3. Cdk2 is required for efficient intra-S checkpoint activation. FACS analysis of wild-type (WT), Cdk2$^{-/-}$ and two clones (clone 7 and 11) expressing Cdk2 in Cdk2$^{-/-}$ HCT-116 cells in hydroxyurea (2mM, 20h - HU-0h) and 6 hrs after release from the G1/S block (6h). NT, non-treated cells. Related checkpoint response is shown in Figure 1d.

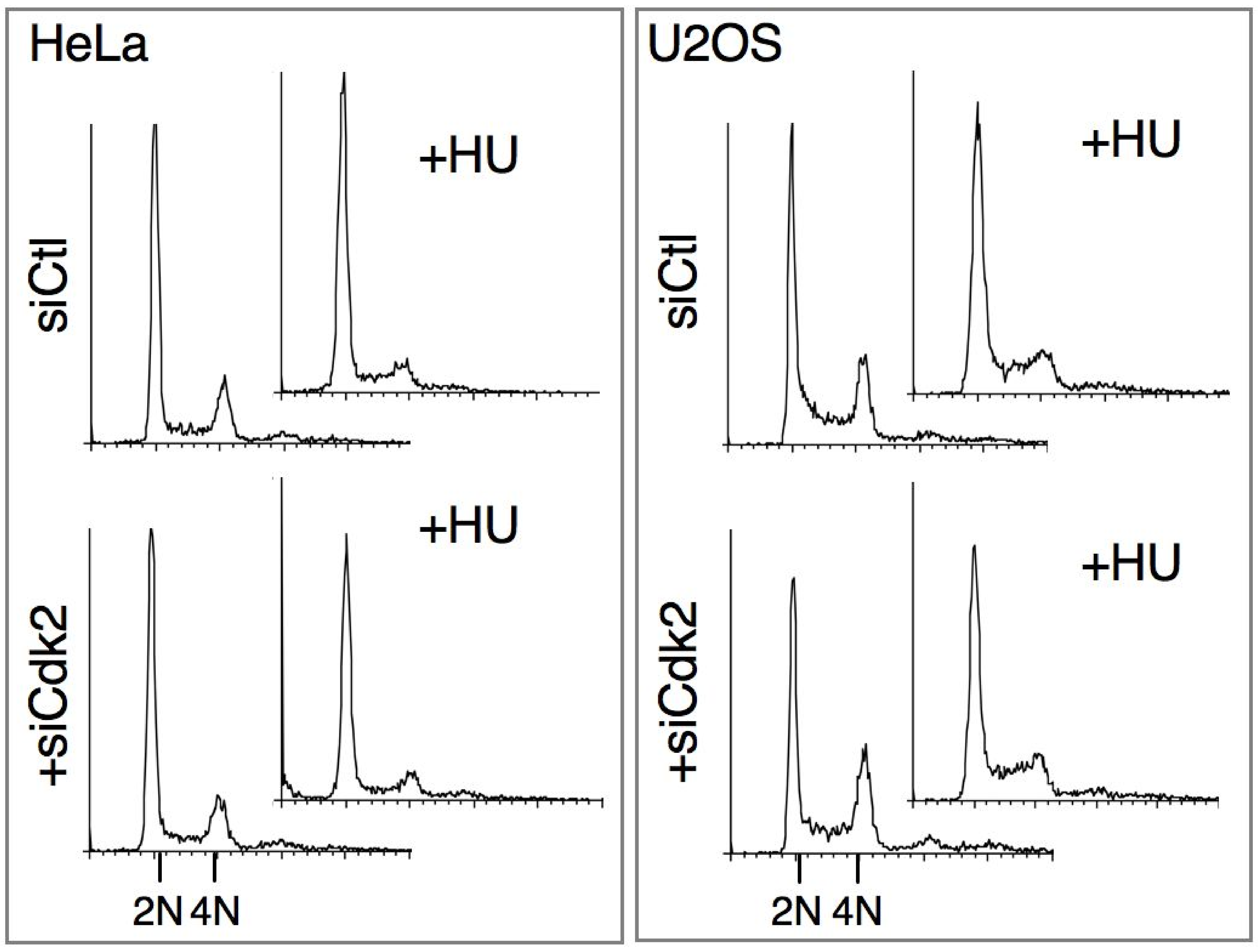

Supplementary Figure S4. Cdk2 is required for efficient intra-S checkpoint activation. FACS analysis of non-treated (NT) and HU-arrested control (siCtl) and Cdk2 knockdown (siCdk2) HeLa and U2OS cells analyzed for the checkpoint response in Figure 1e.

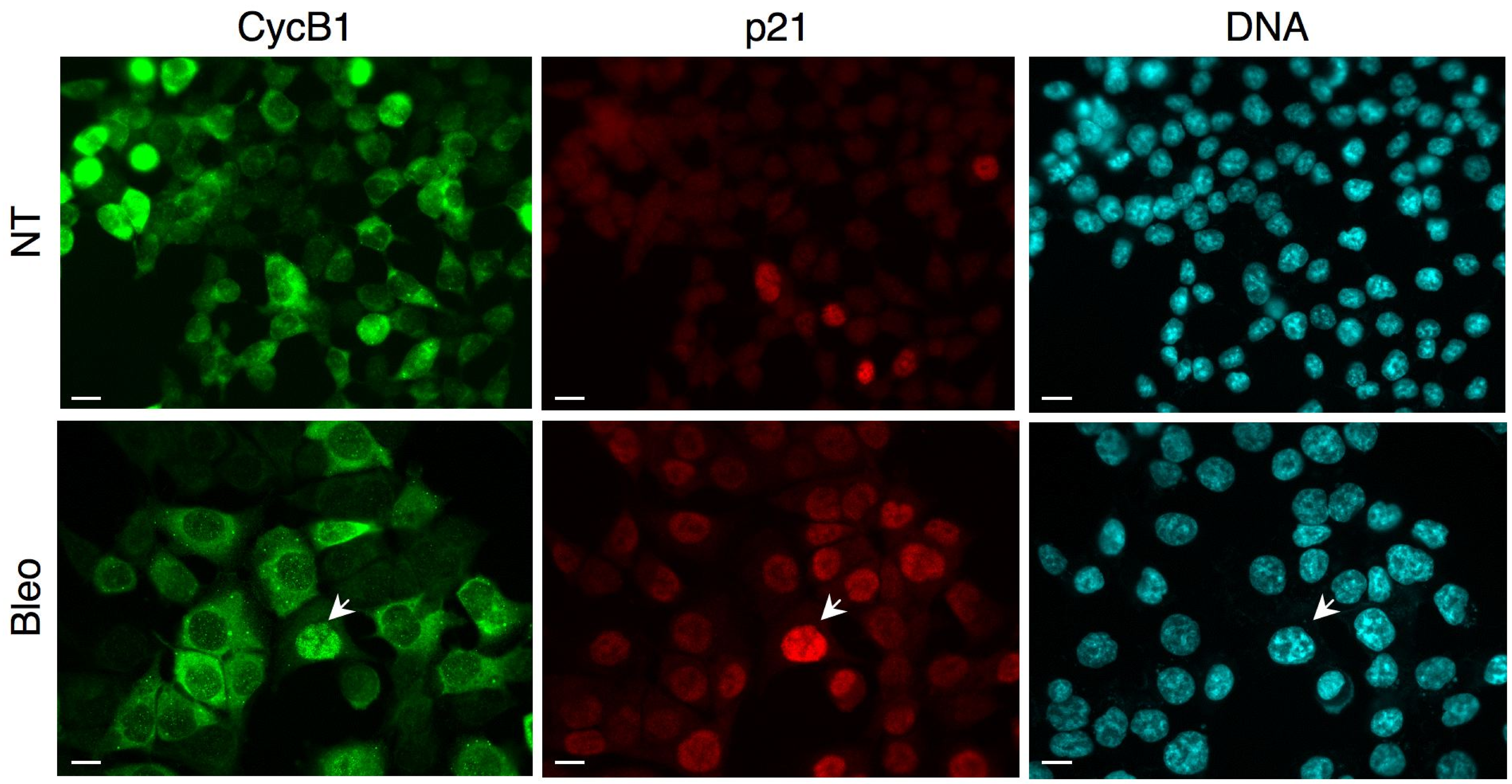

Supplementary Figure S5. CycB1-Cdk1 is not a major target of p21 in HCT-116 cells. Immunofluorescence showing co-staining of CycB1 and p21 in non-treated (NT) and HCT-116 cells exposed to bleomycin (24h). Arrow indicates a cell arrested in G2 with nuclear CycB1 overexpressing p21. Bar, 10 μM.

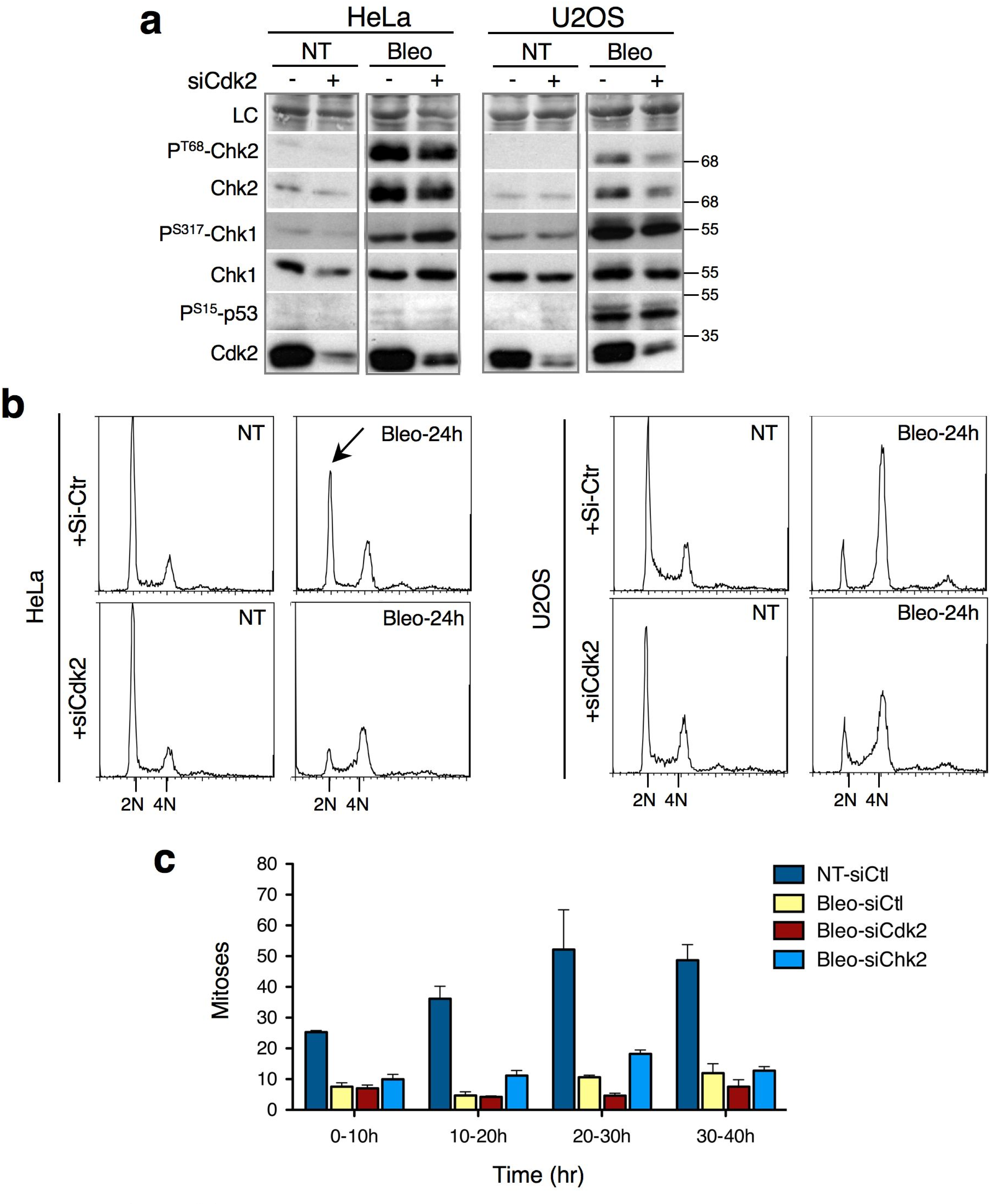

Supplementary Figure S6. Lack of Cdk2 delays S-G2 progression upon DNA damage but does not impair Chk1 activation.

a. Immunoblots showing DNA damage response in control (siCtl) and Cdk2 knockdown (siCdk2) HeLa and U2OS cells exposed to bleomycin for 24h. NT, non-treated cells.

b. FACS analysis of control (siCtl) and Cdk2 knockdown (siCdk2) HeLa and U2OS cells exposed to bleomycin for 24h. Arrow shows a G1 population in bleomycin-treated HeLa cells that escaped from G2 arrest.

c. Video-microscopy data showing entry into mitosis in bleomycin-treated control (siCtl), Cdk2 knockdown (siCdk2) and Chk2 knock-down (siChk2) HeLa cells. Mitoses were counted at 10 hours interval. Mean and mean deviation of three separate experiments are given.

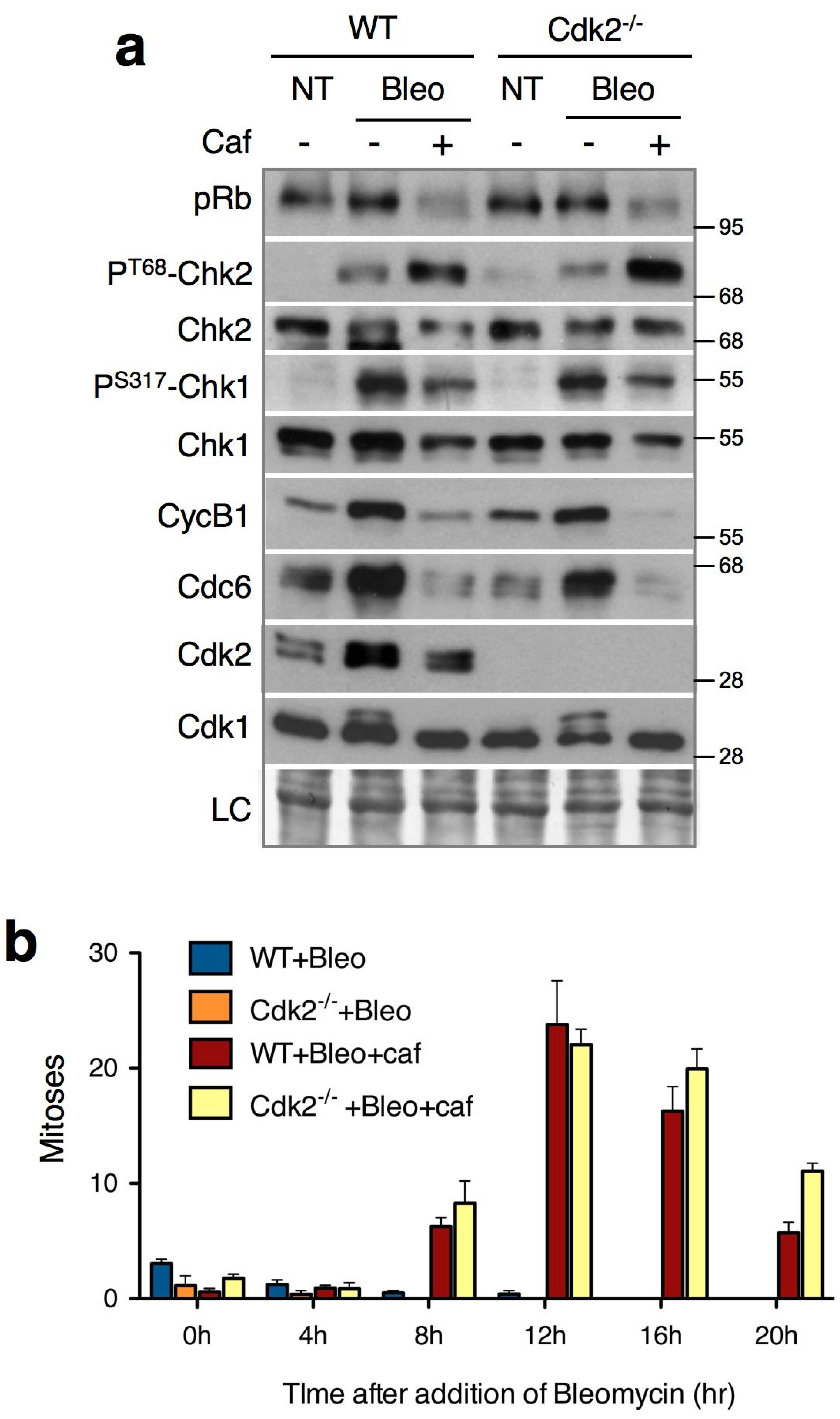

Supplementary Figure S7: Caffeine abrogates G2 arrest by bleomycin in Cdk2$^{-/-}$ HCT-116 cells.
a. Immunoblot analysis showing DNA damage response and indicated cell cycle regulators in wild-type (WT) and Cdk2$^{-/-}$ HCT-116 cells exposed to bleomycin (Bleo, 24h) in the absence (-) or presence of caffeine (caf,+). NT, non-treated cells. LC, loading control.
b. Video-microscopy data showing abrogation of bleomycin-induced G2 arrest by caffeine in wild-type (WT) and Cdk2$^{-/-}$ cells HCT-116 cells. Mitoses were scored at 4 hours interval. Mean and mean deviation of three separate experiments are given.

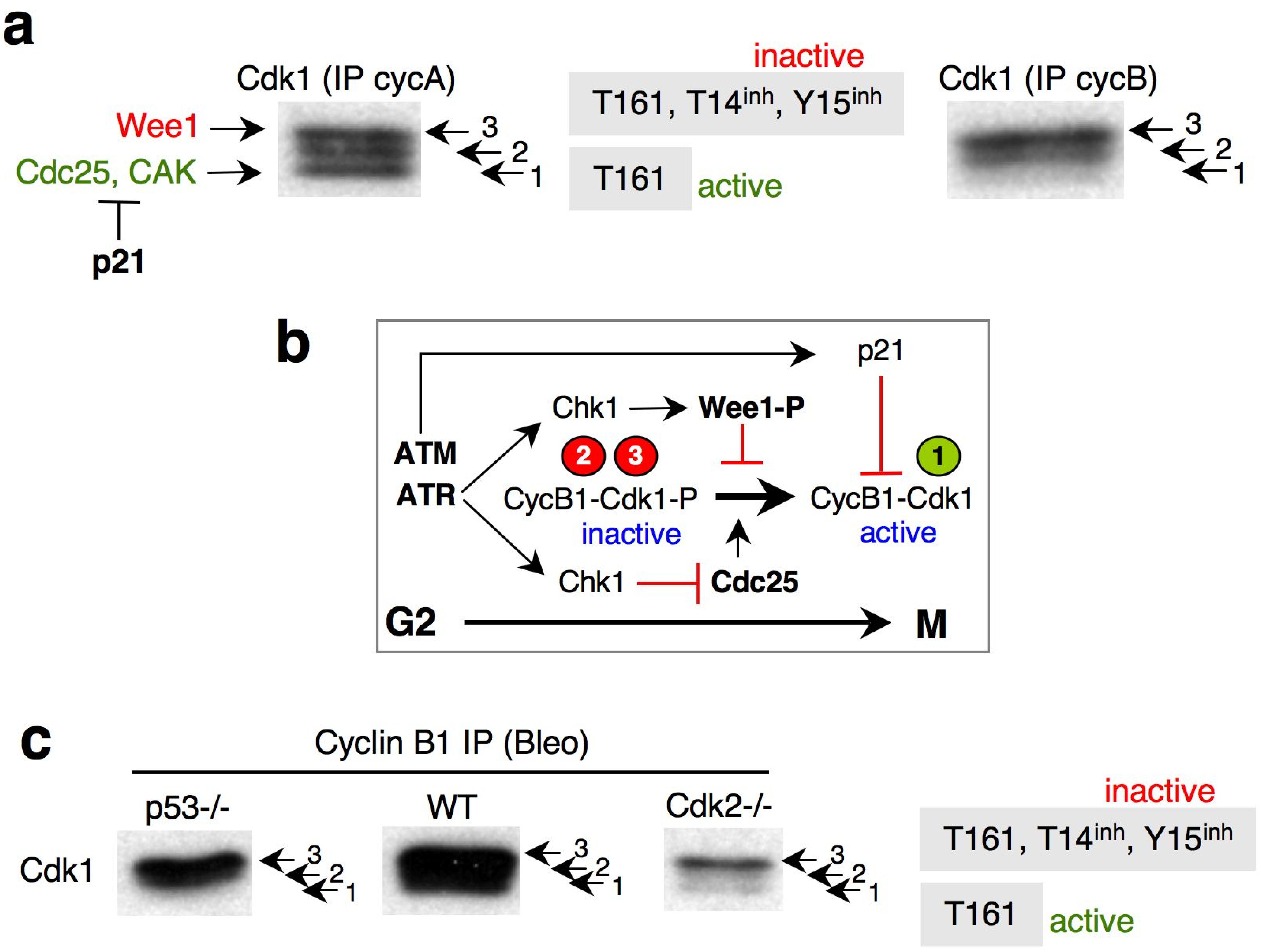

Supplementary Figure S8. Regulation of Cdk1 activity by phosphorylation.

a. Immunoblots showing Cdk1 phospho-isoforms in CycA and CycB1 immunoprecipitates (IP). Numbers 1,2 and 3 denote SDS-PAGE bands of different Cdk1 phospho-isoforms generated by the actions of Cdk-activating kinase (CAK; T161), CDK inactivating kinase Wee1 ($T14^{inh}$, $Y15^{inh}$) and Cdc25 phosphatases (see b for explanation).

b. Model showing DNA damage signalling network leading to inhibition of CycB1-Cdk1 complexes.

c. Immunoblots showing Cdk1 phospho-isoforms in CycB1 immunoprecipitates (CycB1 IP) isolated from wild-type (WT), p53-/-, WT and Cdk2-/- HCT-116 cells exposed to bleomycin (24h). Numbers 1,2 and 3 denote different Cdk1 phospho-isoforms.

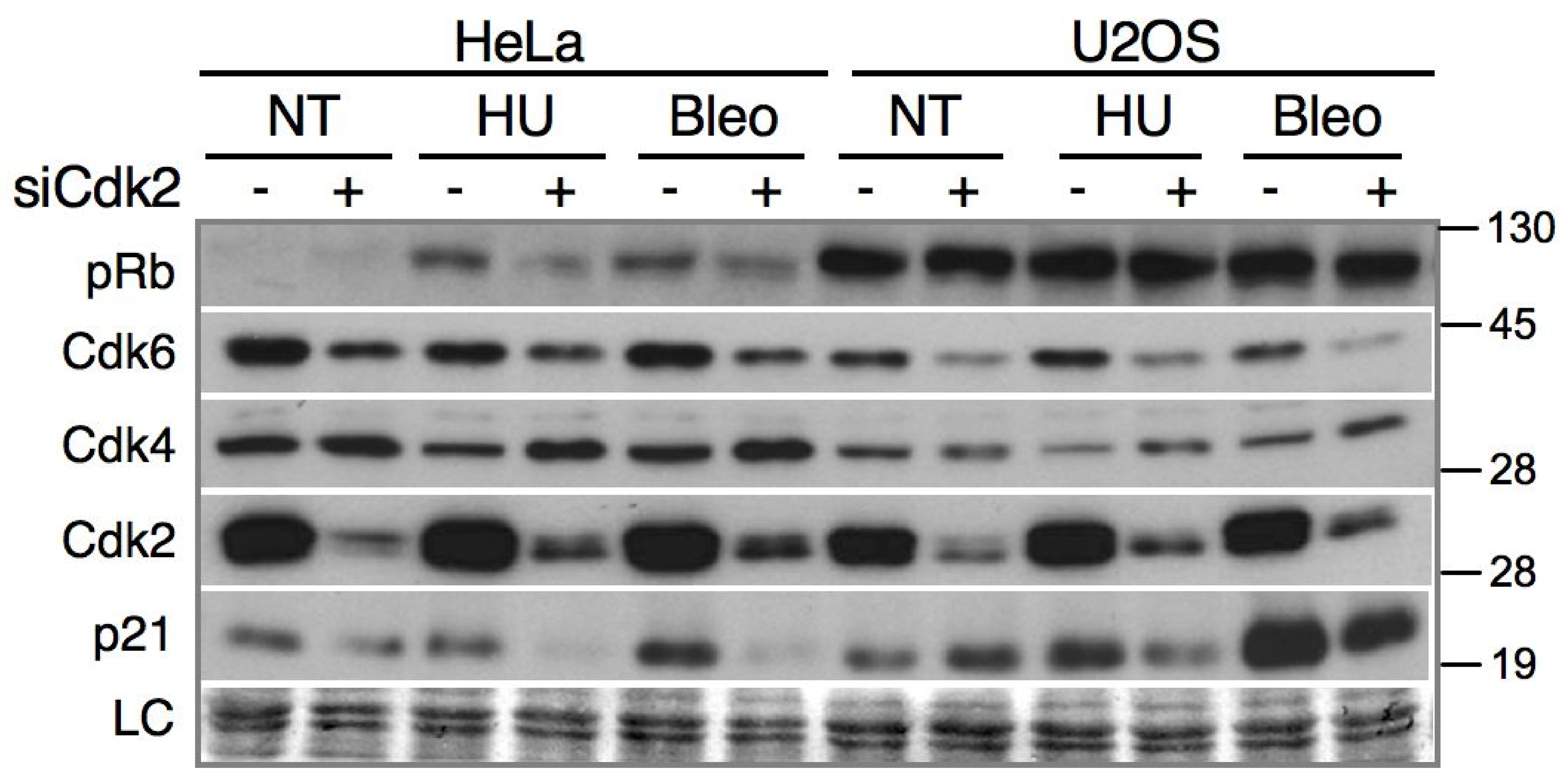

Supplementary Figure S9. Cdk2 controls Cdk6 levels. Immunoblot analysis showing the effect of Cdk2 knockdown (siCDk2) on the components of pRb pathway in HeLa and U2OS cells exposed to hydroxyurea (HU, 20h) and bleomycin (Bleo, 24h). NT, non-treated cells. LC, loading control.

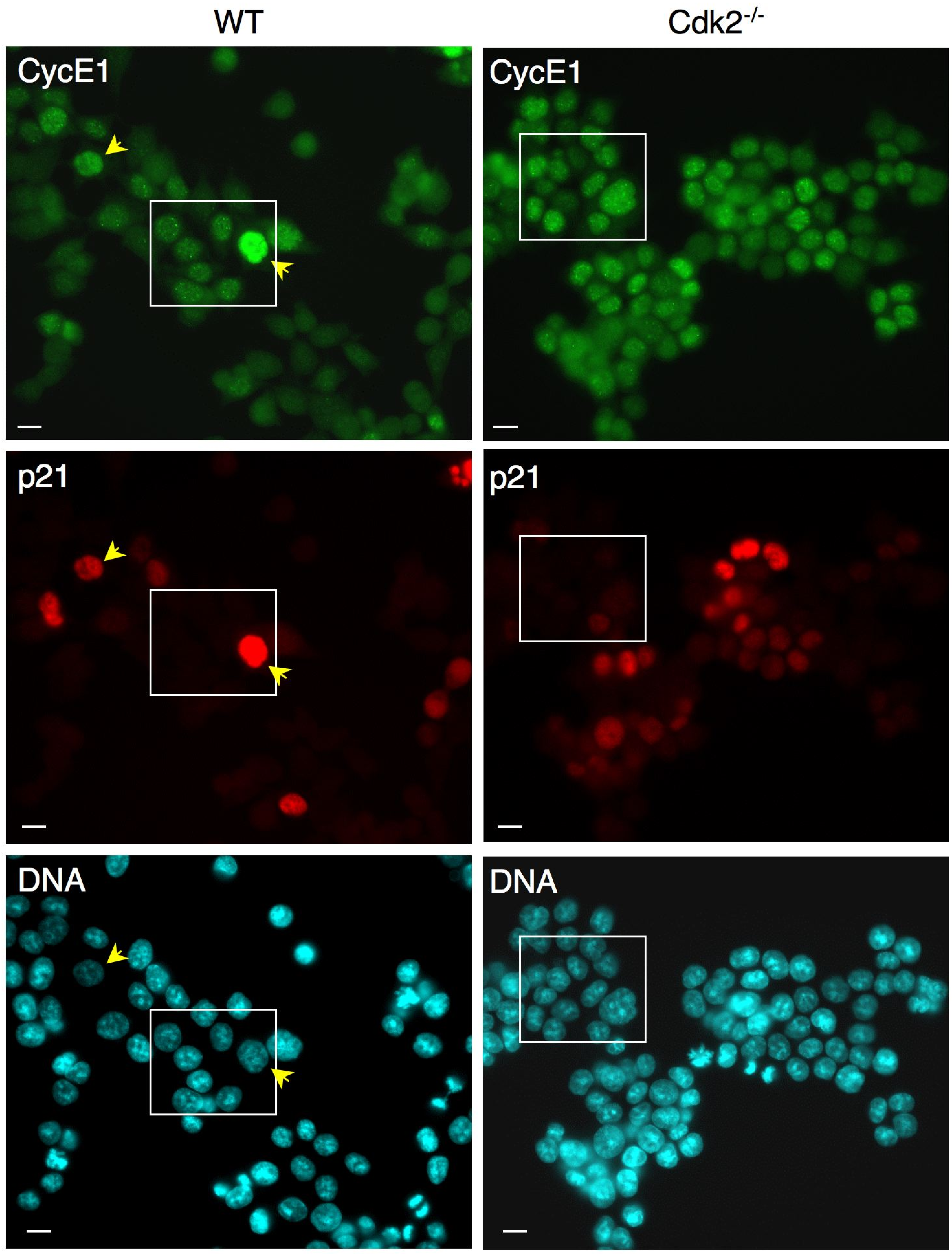

Supplementary Figure S10. Cyclin E1-Cdk2 is a preferential p21 target in DNA damage-induced cell cycle exit

Immunofluorescence showing co-staining of CycE1 and p21 in proliferating WT and $Cdk2^{-/-}$ HCT-116 cells. Arrows indicate apparently senescent cells that accumulate p21 and CycE1 and exhibit weak perinucleolar heterochromatin. Rectangles indicate the fields shown in Figure 5a. Bar, 10 μM.

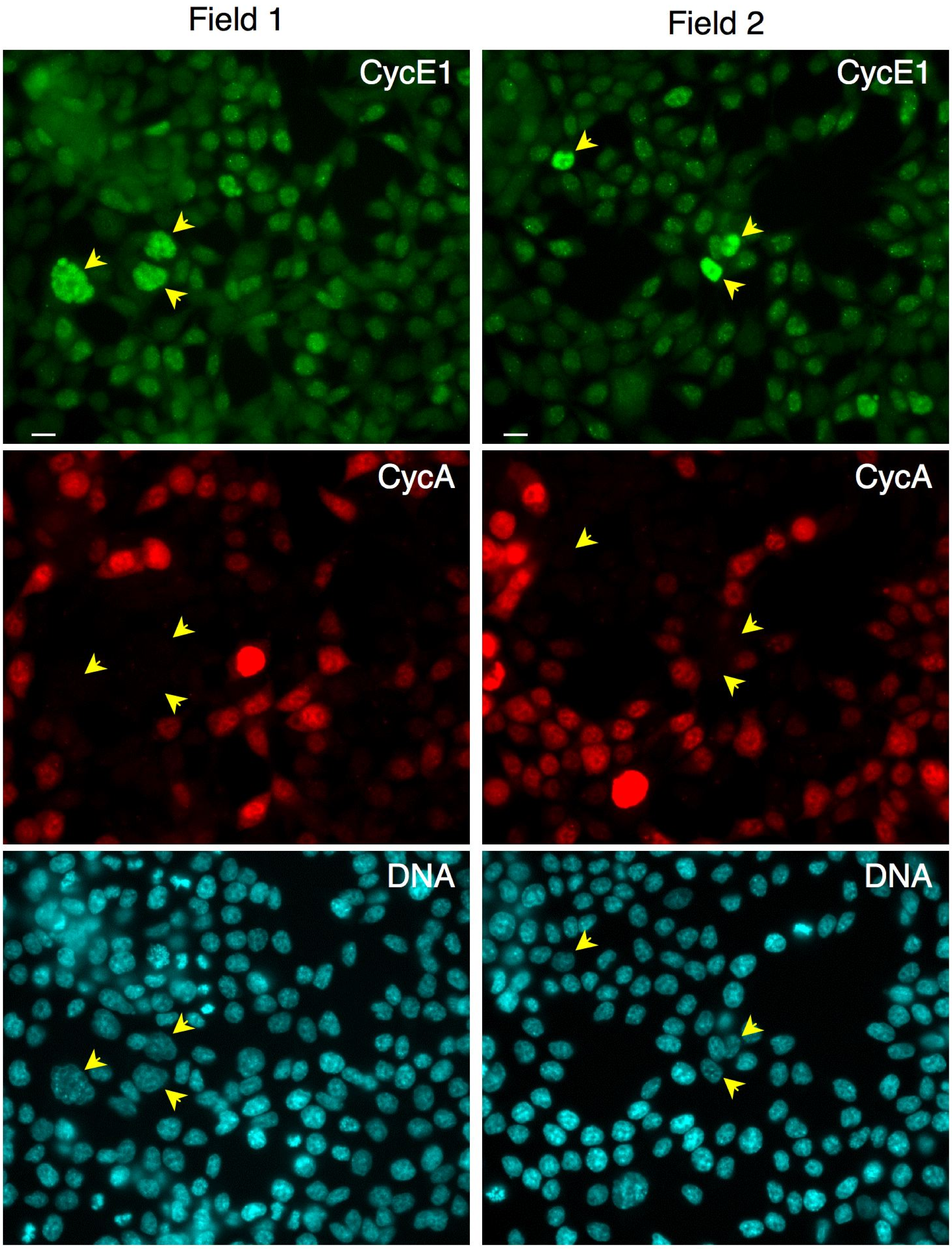

Supplementary Figure S11. The cells over-expressing cyclin E1 do not cycle. Immunofluorescence showing co-staining of CycE1 and CycA in proliferating wild-type HCT-116 cells. Arrows indicate apparent senescent cells overexpressing CycE1 and lacking CycA and perinucleolar heterochromatin. Two representative fields are shown. Bar, 10μM.

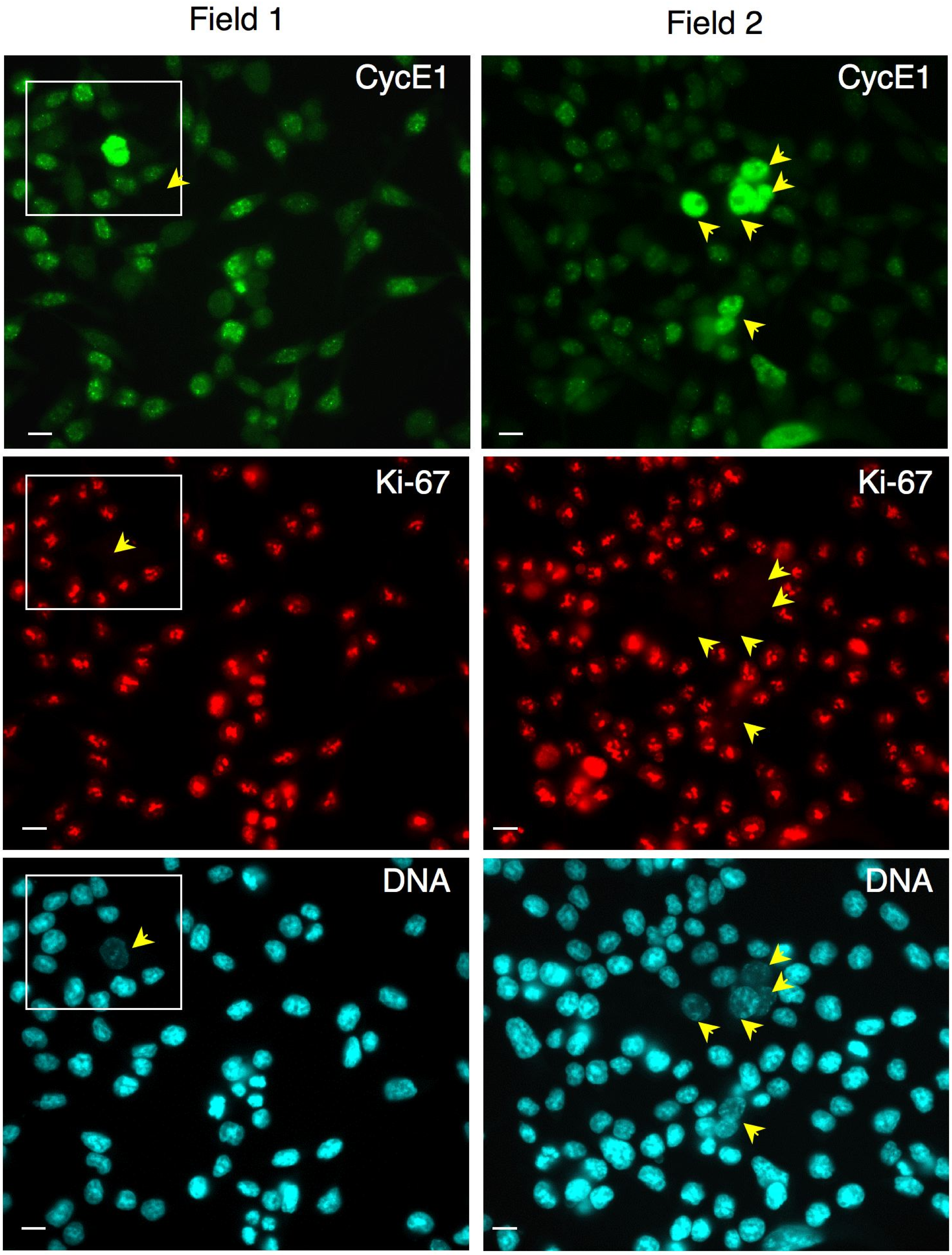

Supplementary Figure S12. Cells expressing high Cyclin E1 levels are senescent. Immunofluorescence showing co-staining of CycE1 and Ki-67 in proliferating WT HCT-116 cells. Two representative fields are shown. Arrows show apparently senescent cells that accumulate CycE1 and exhibit weak Ki-67 signal. Rectangles indicate the field shown in Figure 5c. Bar, 10 µM.

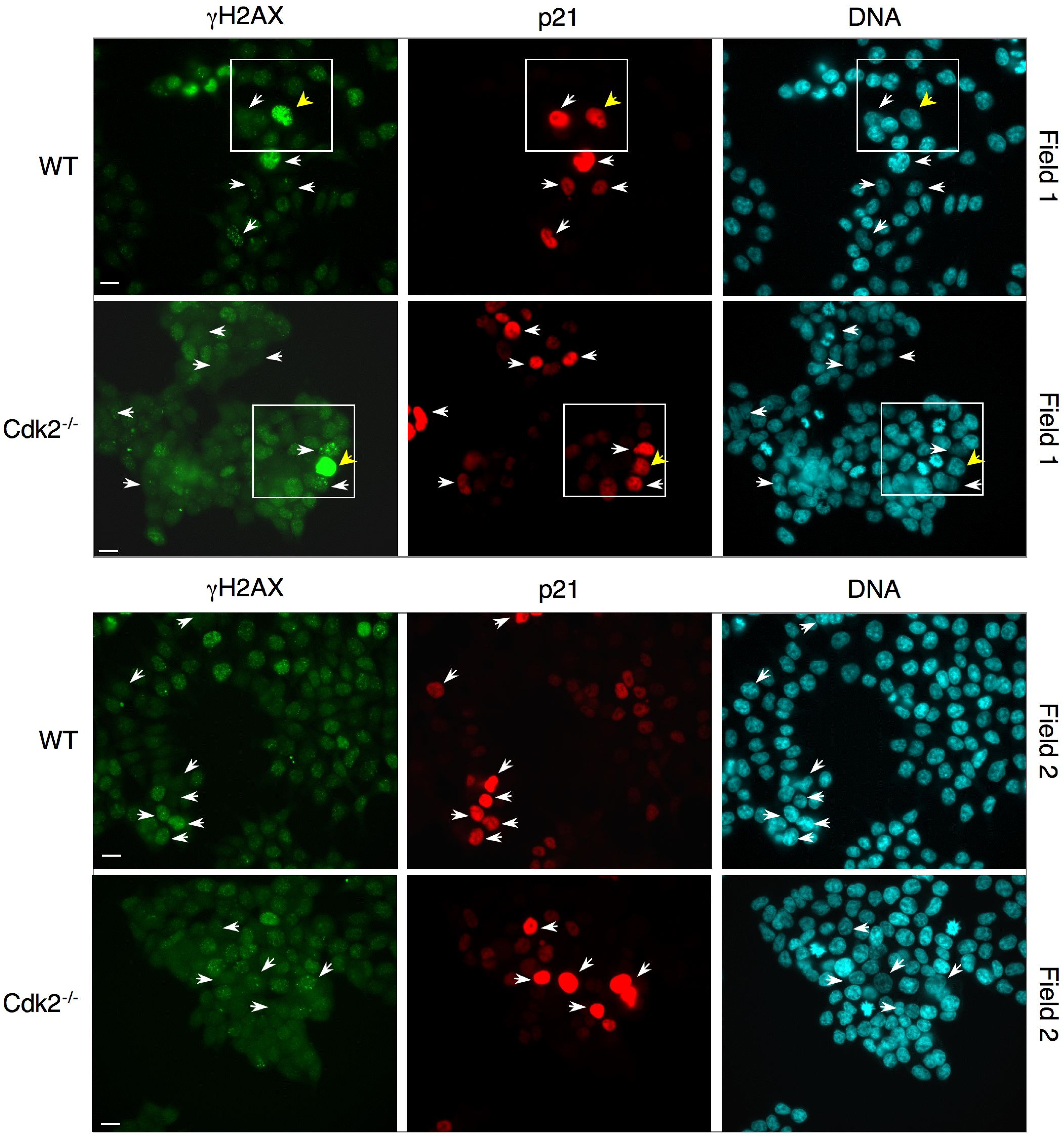

Supplementary Figure S13. Presence of DNA damage and p21 upregulation in subpopulation of proliferating HCT-116 cells.

Immunofluorescence showing γH2AX and p21 co-staining in proliferating HCT-116 cells. Two representative fields are shown. Yellow arrows indicate cells with strong γH2AX signal. White arrows indicate cells with strong p21 signal. Note that p21 upregulation is not always associated with strong γH2AX signal. Rectangles indicate the field shown in Figure 5e. Bar, 10 μM

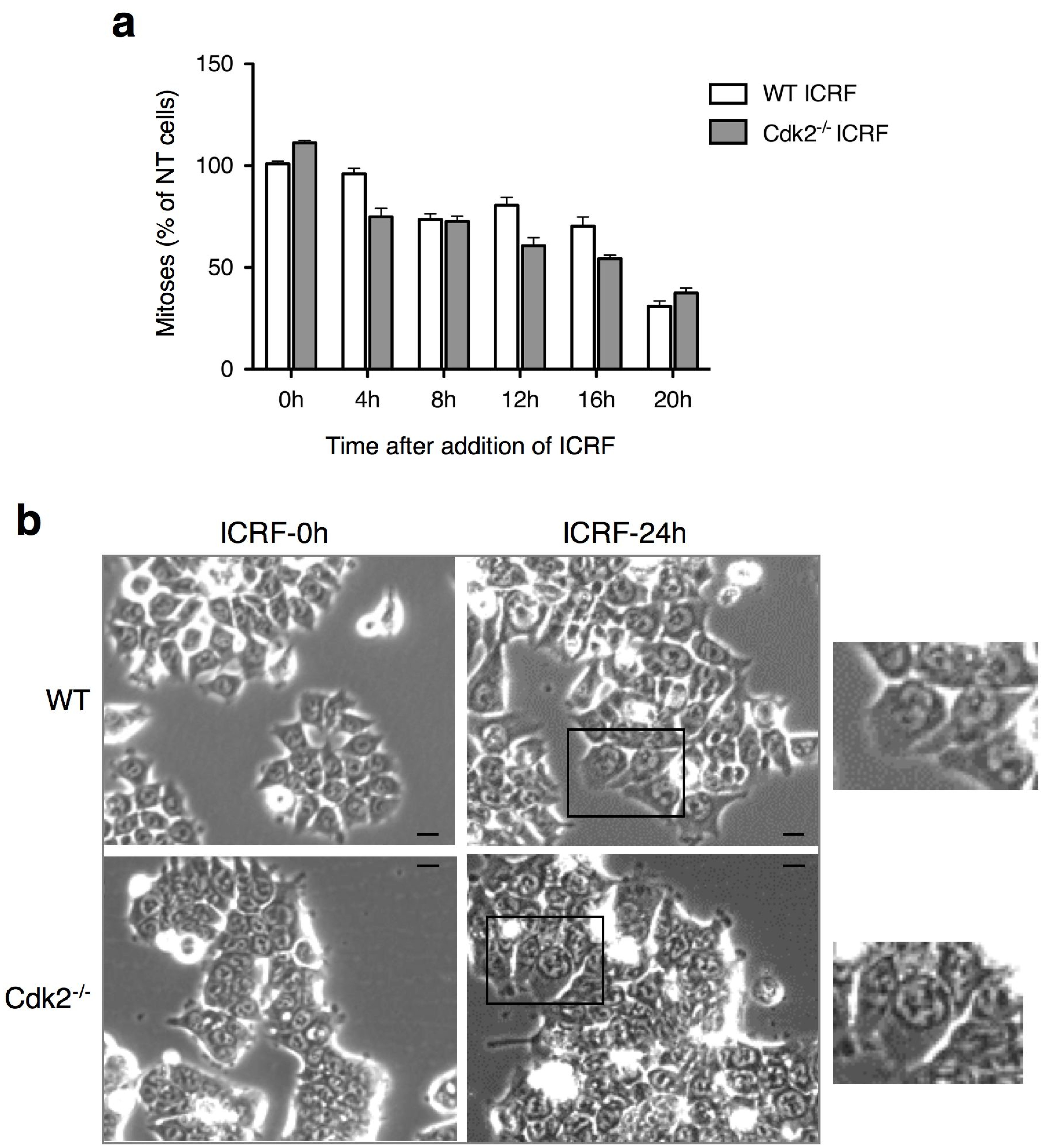

Supplementary Figure S14: ICRF cannot block mitosis in WT and Cdk2$^{-/-}$ HCT-116 cells.

a. Video-microscopy data showing kinetics of mitosis entry in the presence of ICRF in WT and Cdk2KO HCT-116 cells. Mean and mean deviation of three separate experiments are given.

b. Micrographs extracted from the time-lapse films showing aberrant nuclei (rectangle) in ICRF-treated WT and Cdk2-/- HCT-116 cells. The same field is shown at 0h and after 24h. Bar, 10 μM.

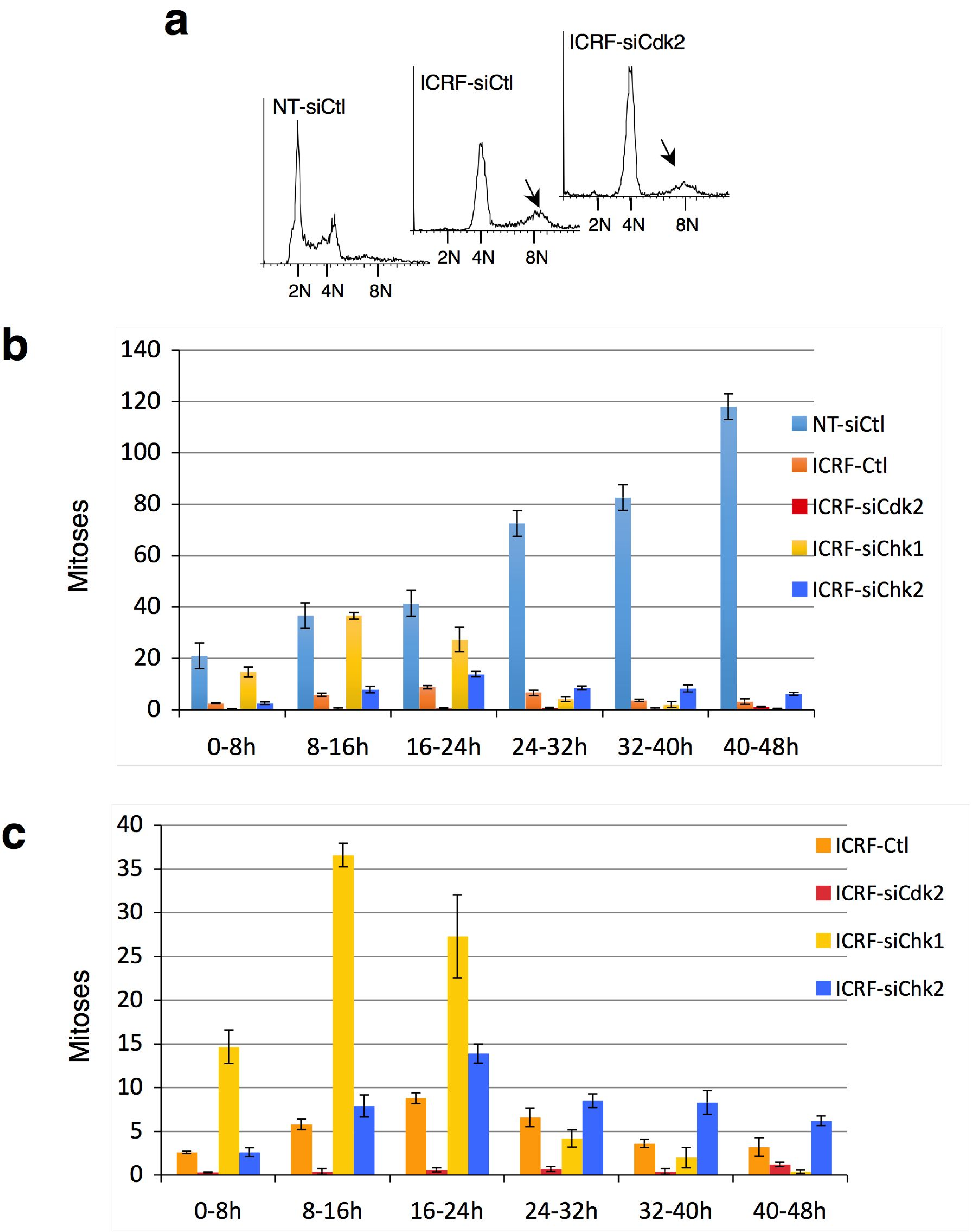

Supplementary Figure S15. Cdk2 depletion prevents mitotic entry upon DNA damage in U2OS cells.

a. FACS analysis showing effects of Cdk2 depletion (siCdk2) in ICRF-treated U2OS cells. Arrows show 8N cells that accumulate in control cells.

b. Video-microscopy data showing occurrence of mitoses in non-treated (NT) and ICRF-treated control (siCtl), Cdk2 KD (siCdk2) and Chk1 KD (siChk1) and Chk2 (siChk2) U2OS cells. Mitoses were counted at 8-hour intervals.

c. The same as in **b** except the NT-siCtl cells were not shown to appreciate better the "mitotic leak" in ICRF-treated cells. Mean and mean deviation of three separate experiments are given.

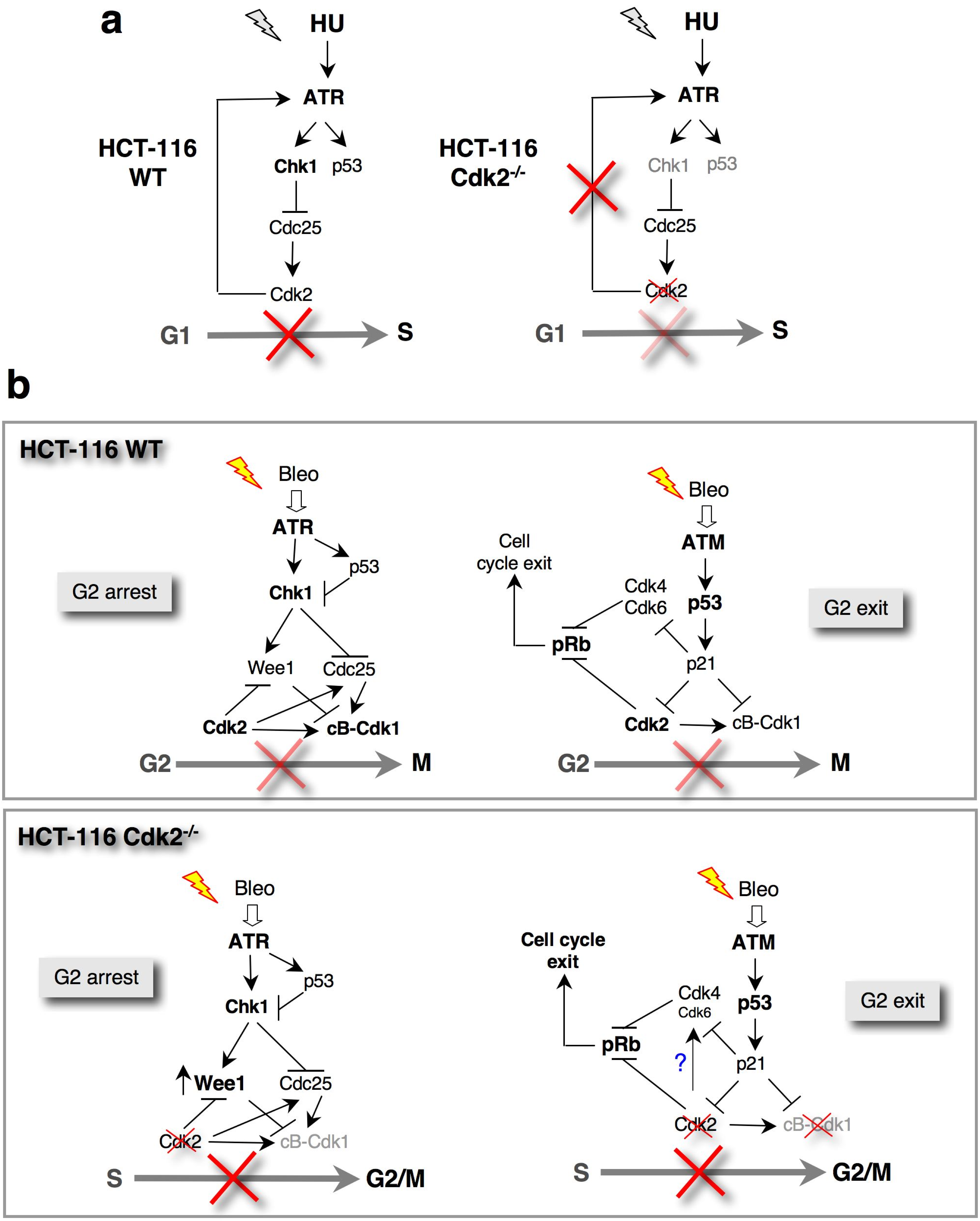

Supplementary Figure S16. A proposed model to explain the phenotype of Cdk2 knockdown (Cdk2$^{-/-}$) in (**a**) intra-S and (**b**) DNA damage-induced G2/M checkpoint in wild type (WT, top) and Cdk2$^{-/-}$ (bottom) HCT-116 cells.

Supplementary Figure S17

Figure 1a

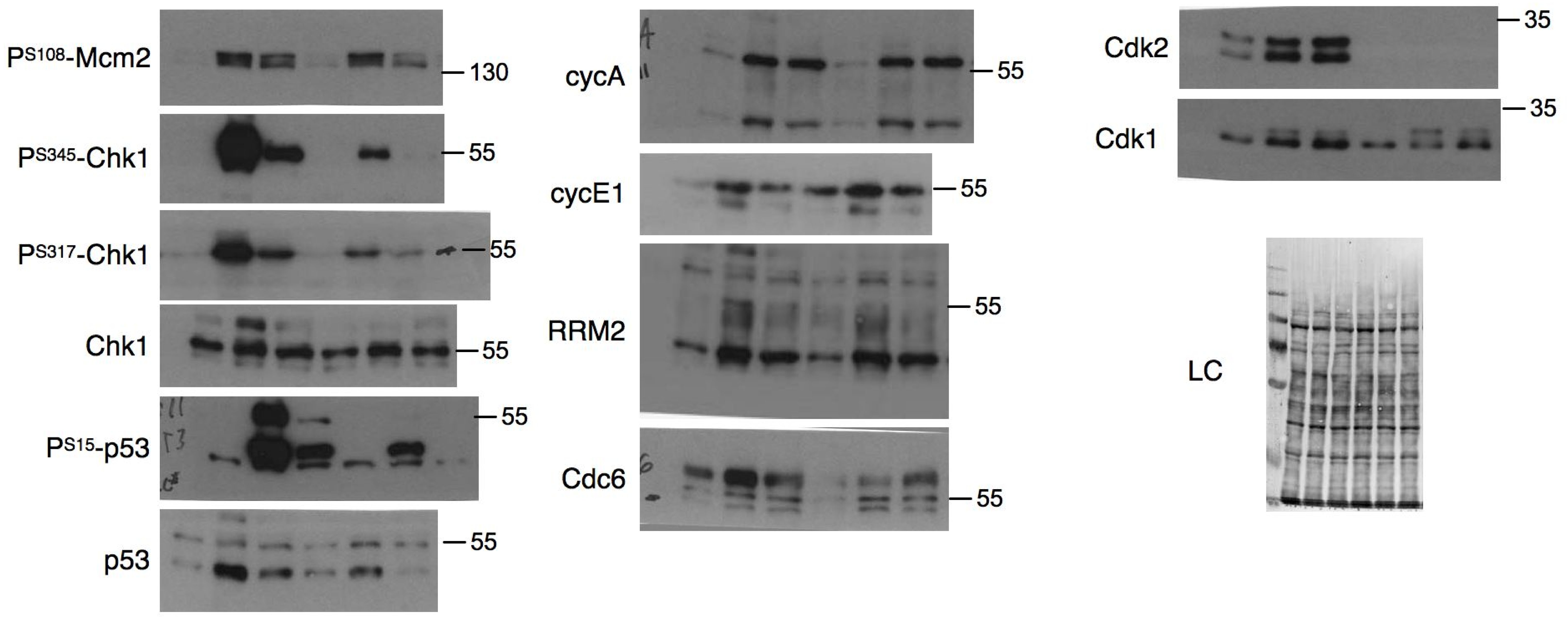

Figure 1d

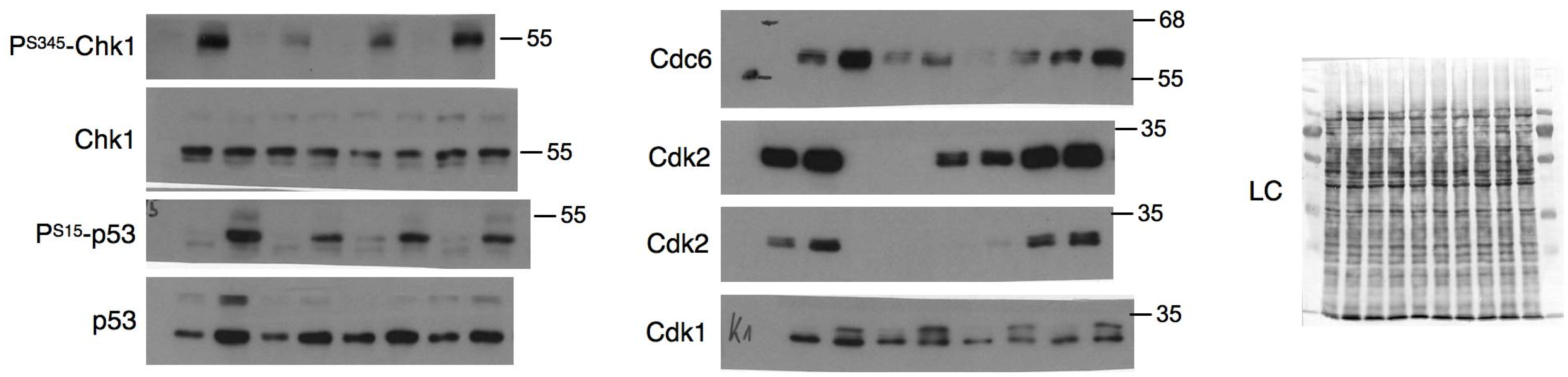

Figure 1e

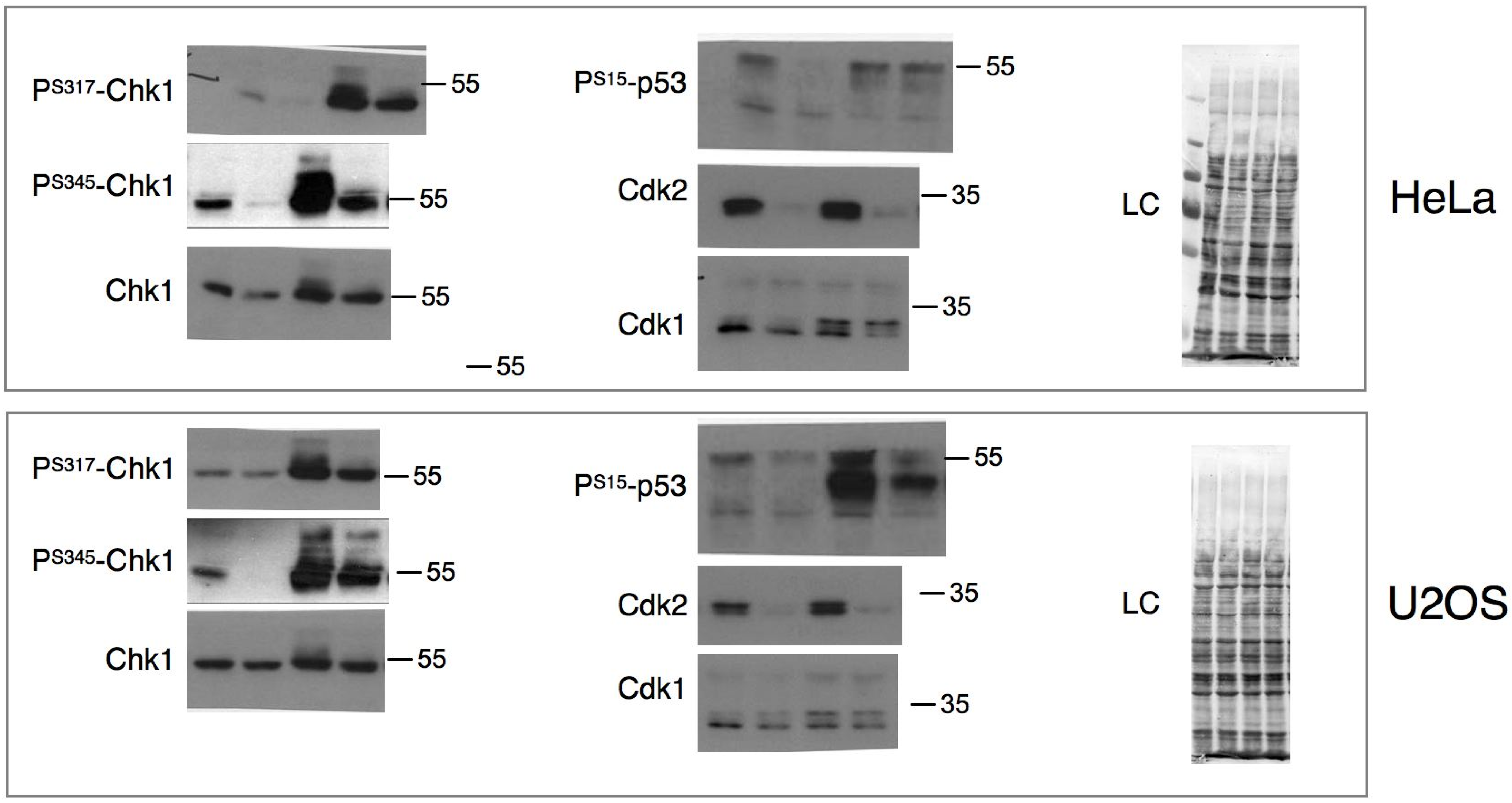

Supplementary Figure S17. Uncropped version of the western blots presented in the main figures.

Figure 2b

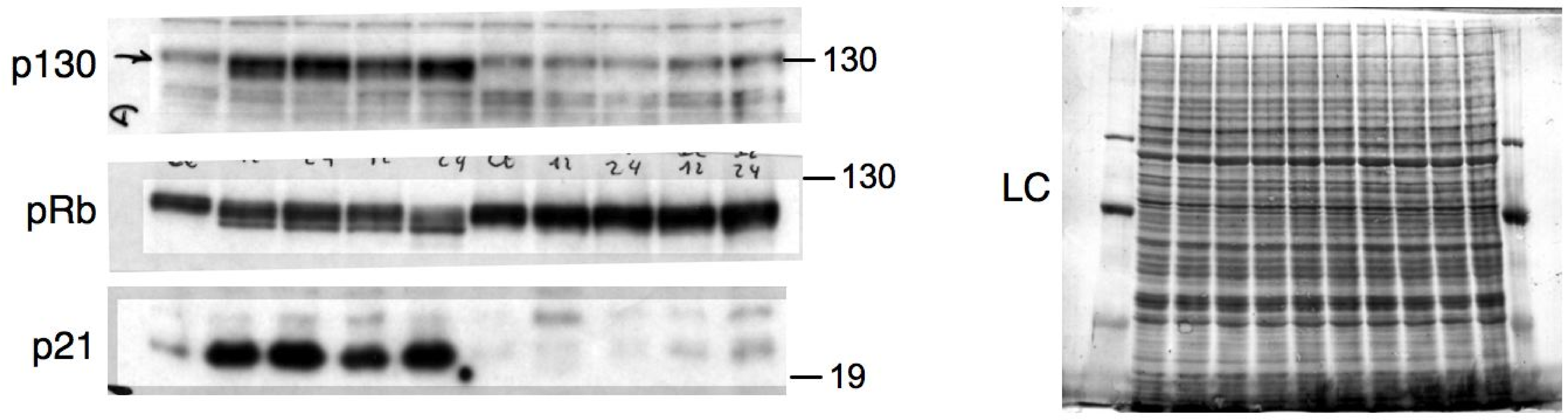

Figure 2c

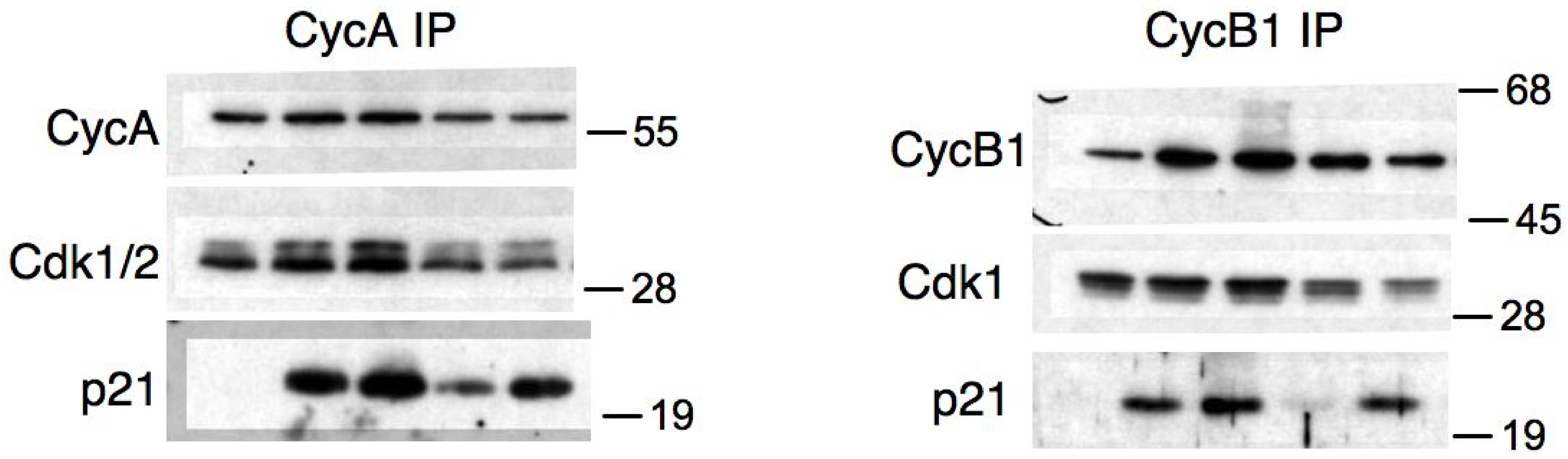

Figure 2d

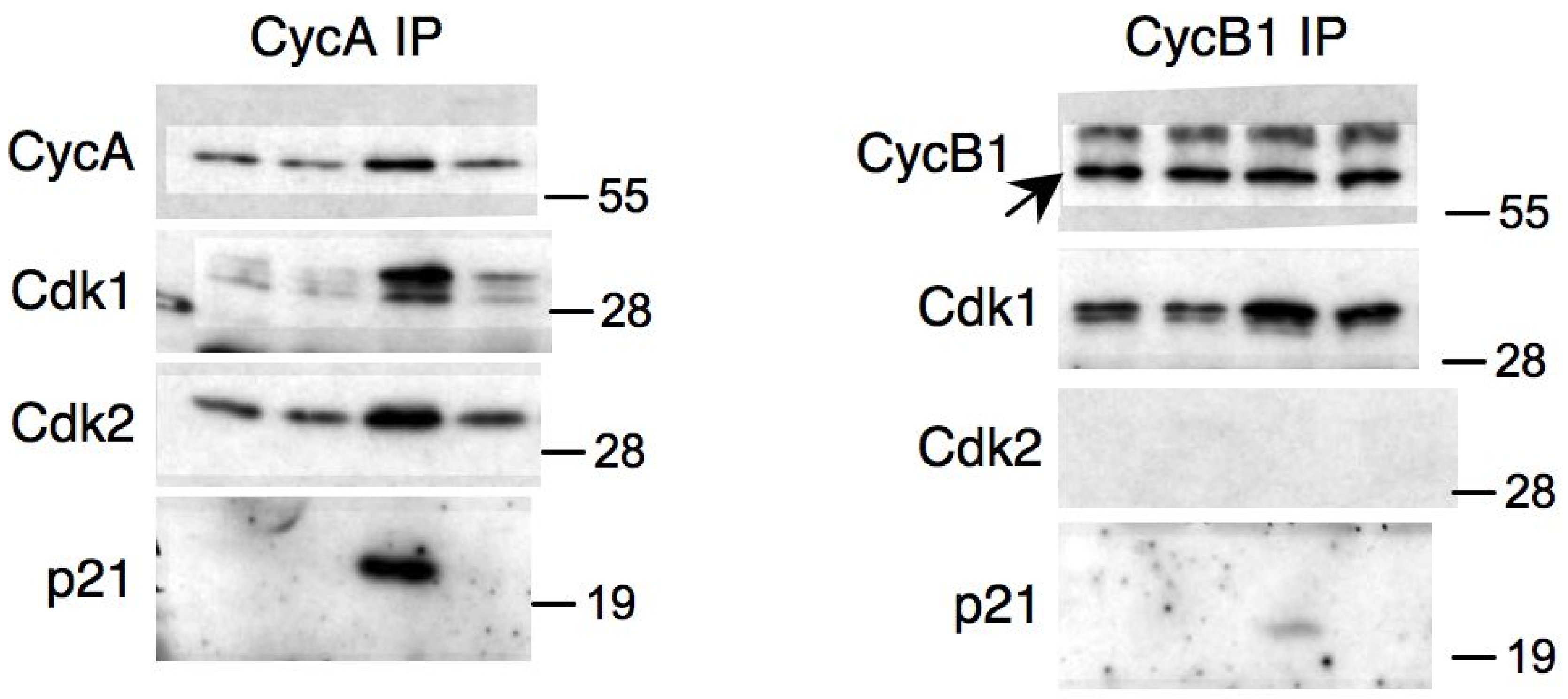

Figure 2e

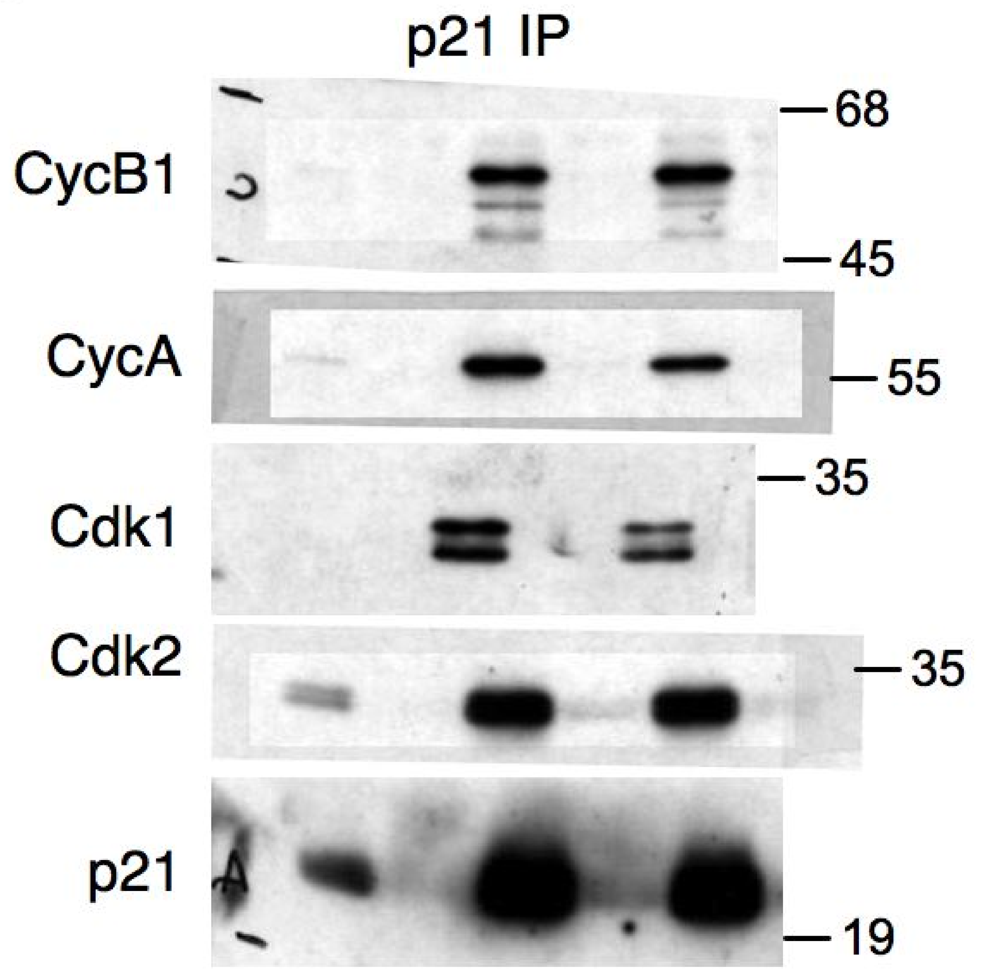

Supplementary Figure S17 continued

Figure 3a

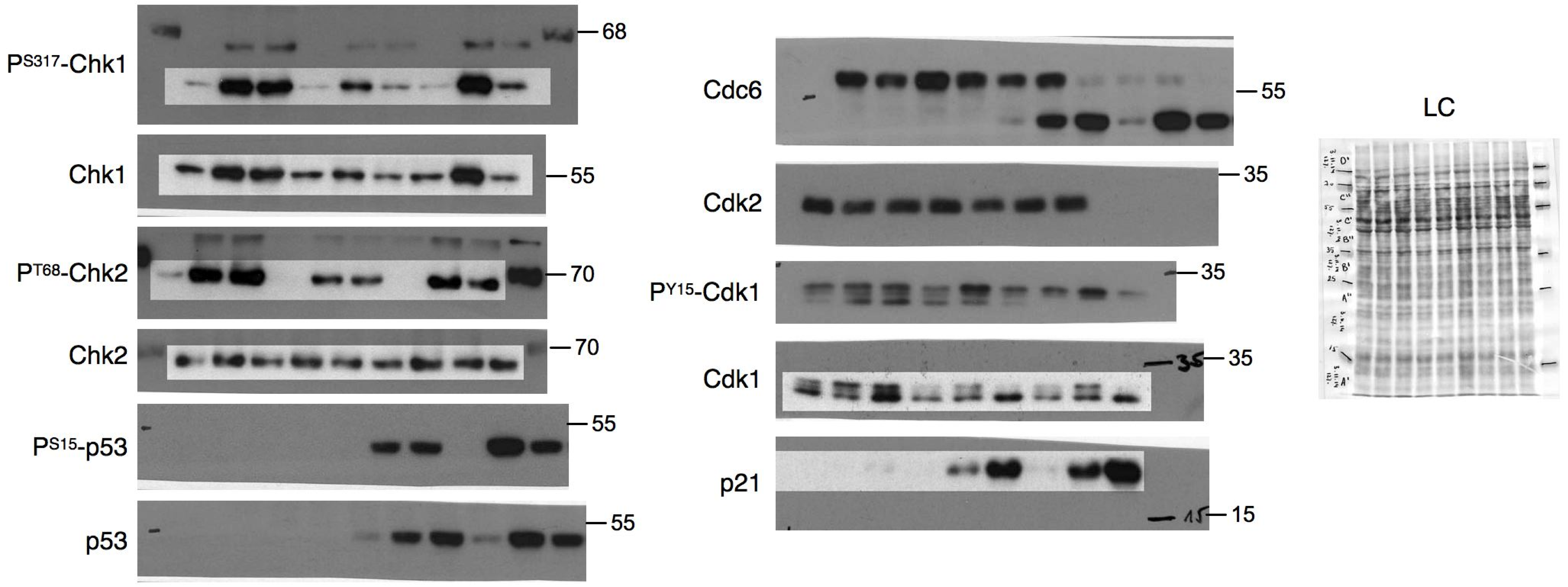

Figure 3c

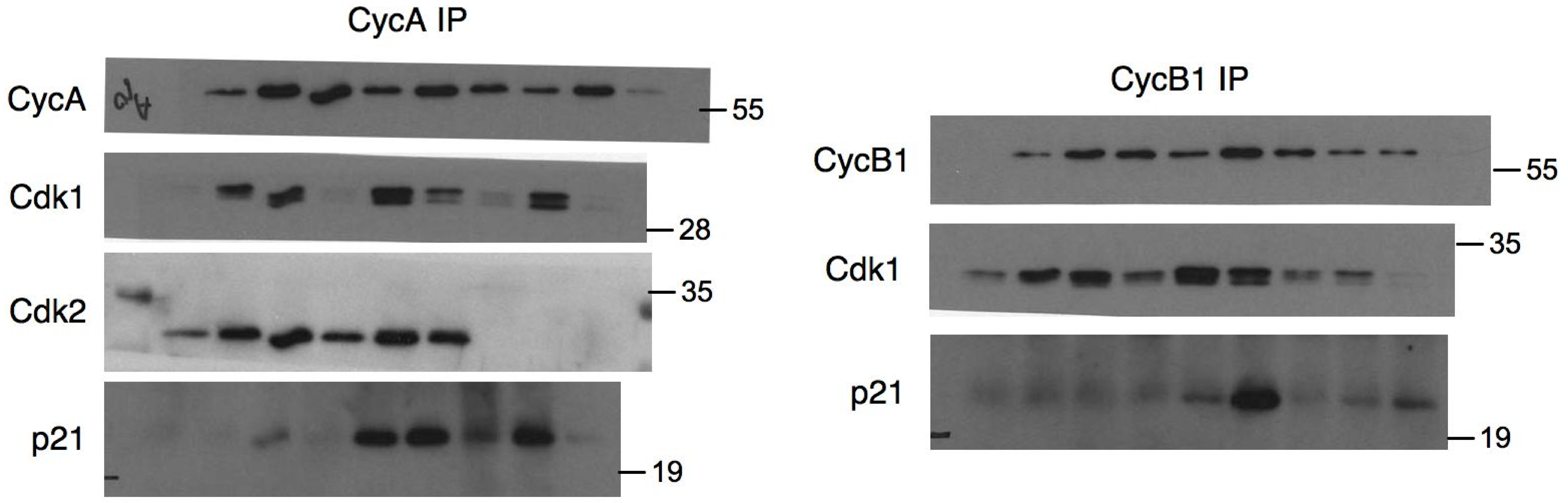

Figure 3d

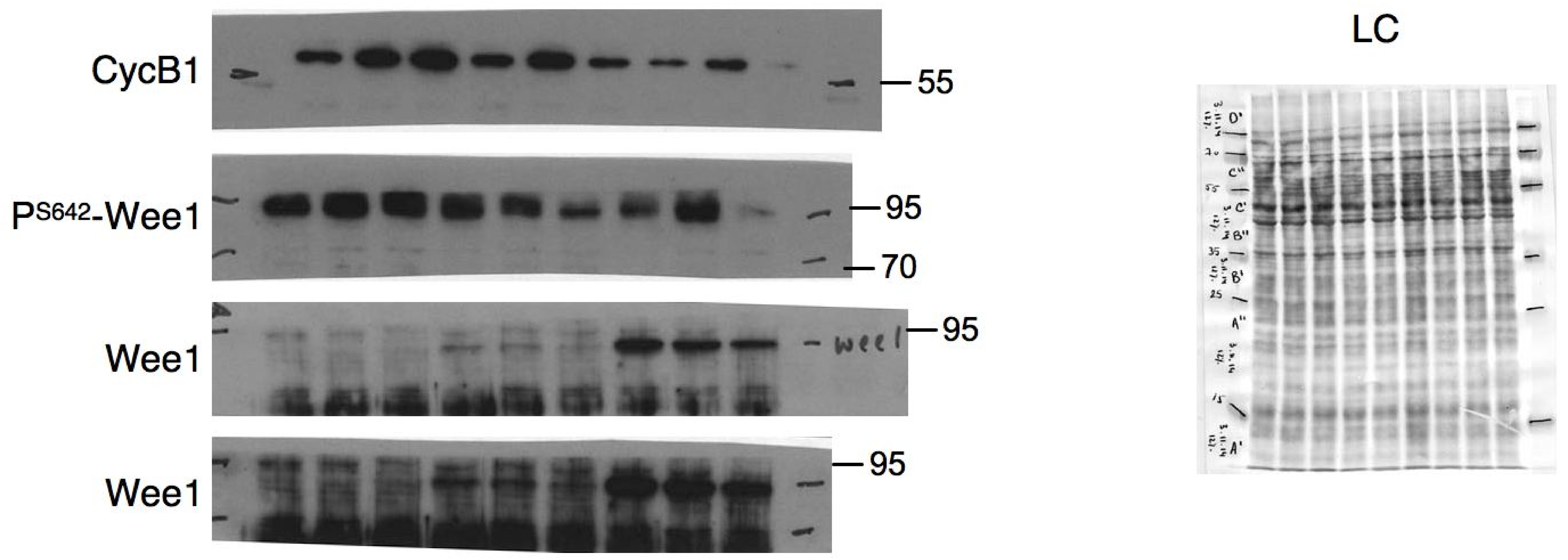

Supplementary Figure S17 continued

Figure 4a

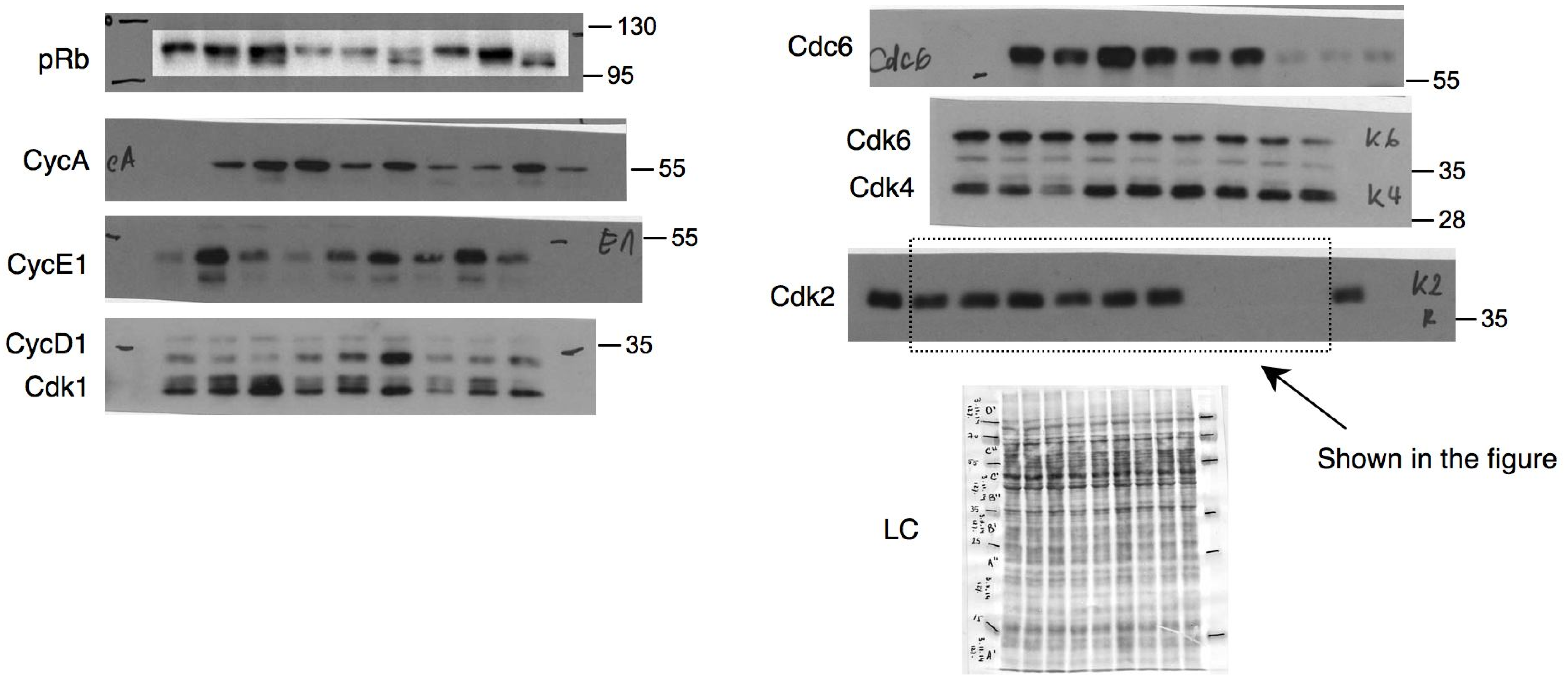

Figure 4b

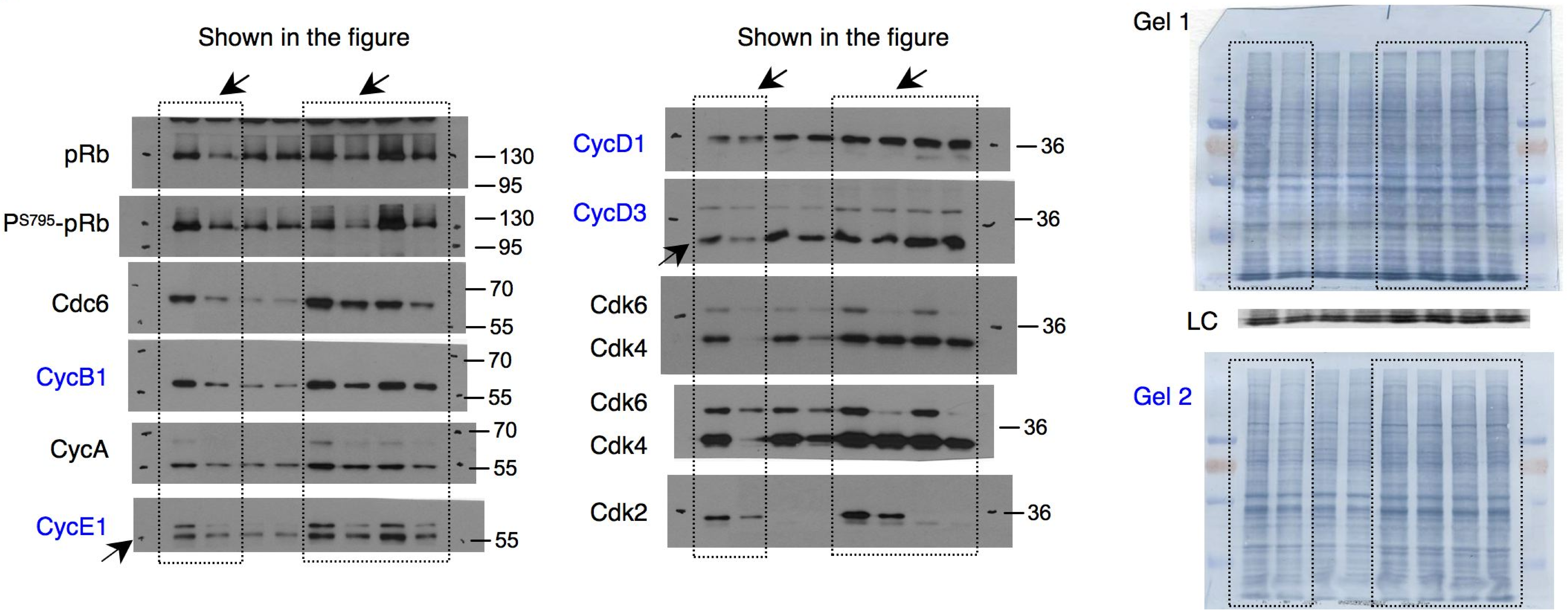

Supplementary Figure S17 continued

Figure 6A

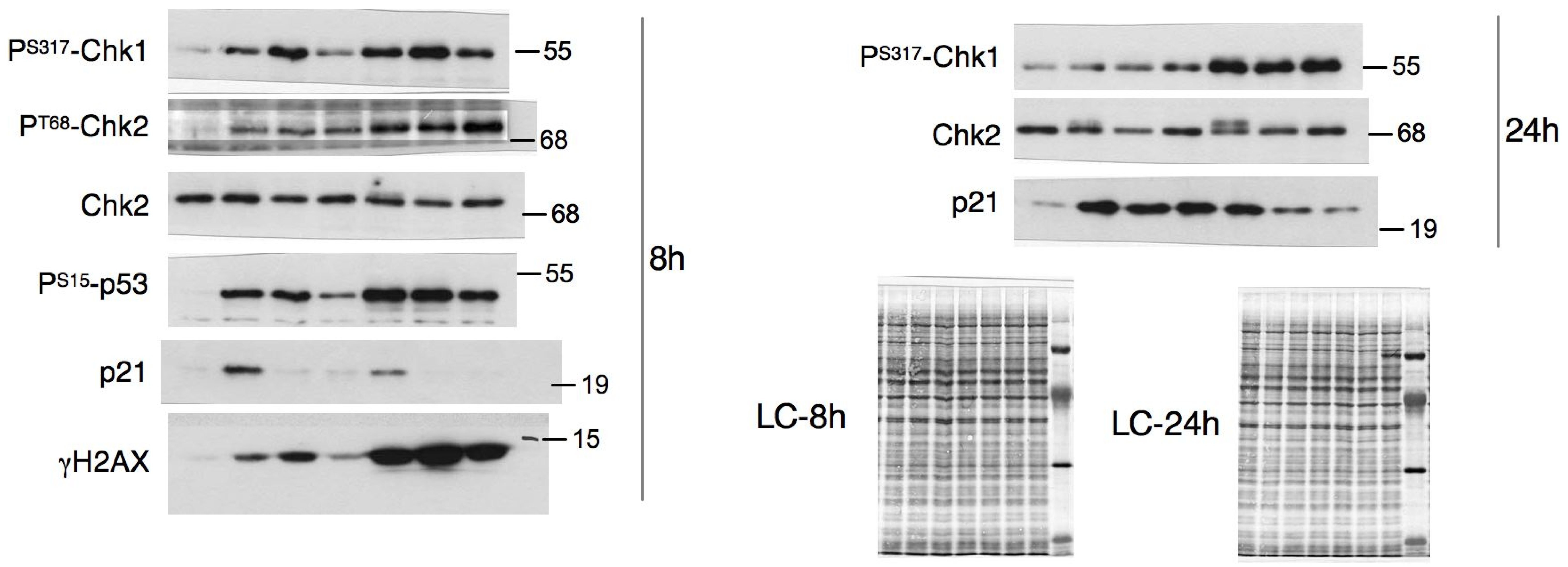

Figure 6d

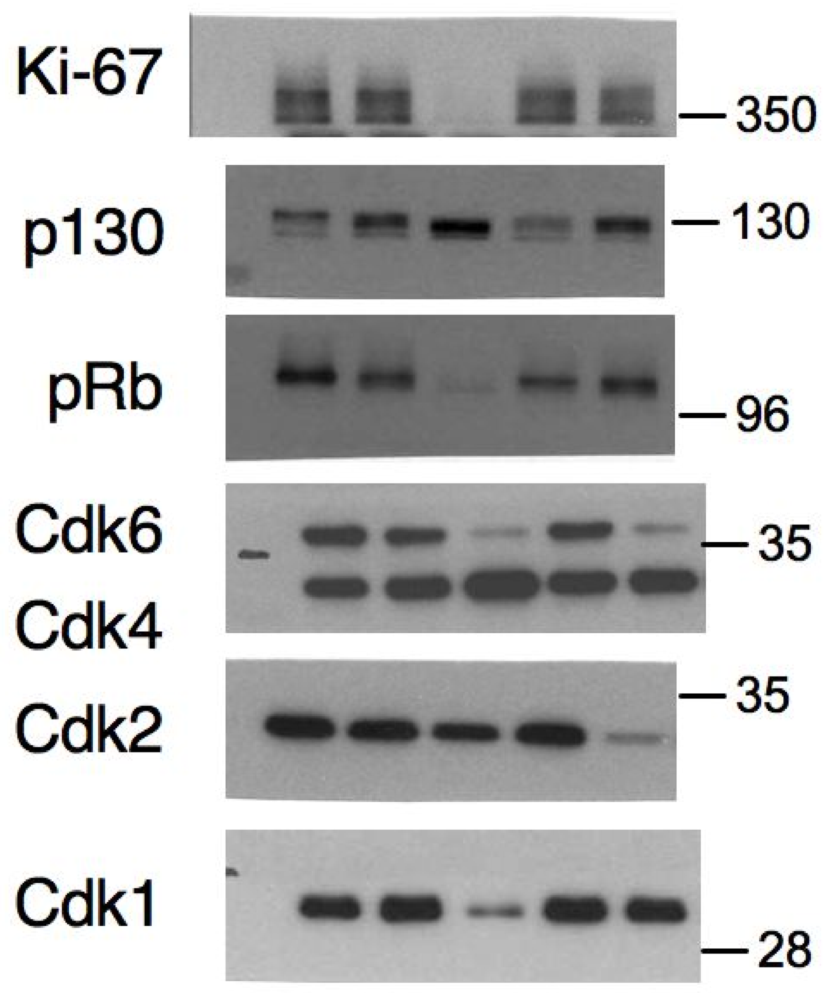

Figure 6e

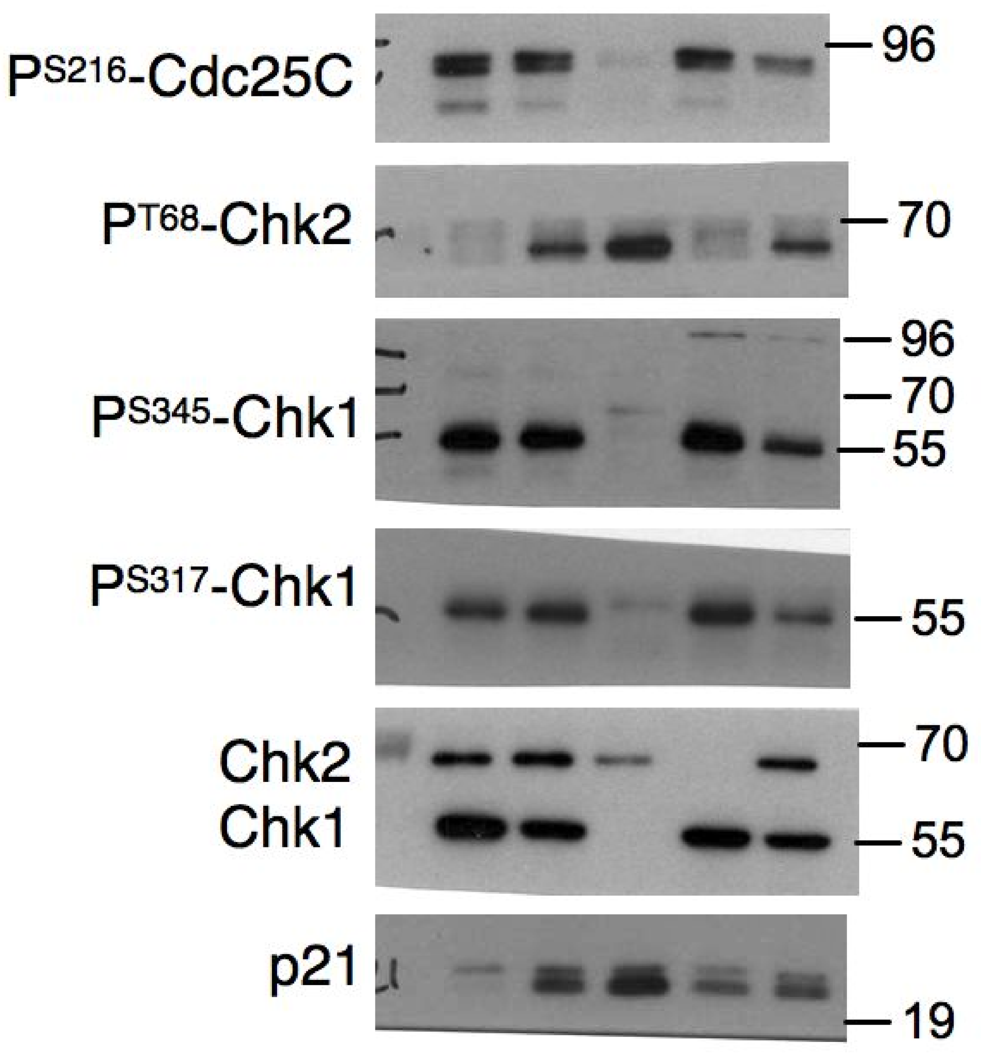

LC

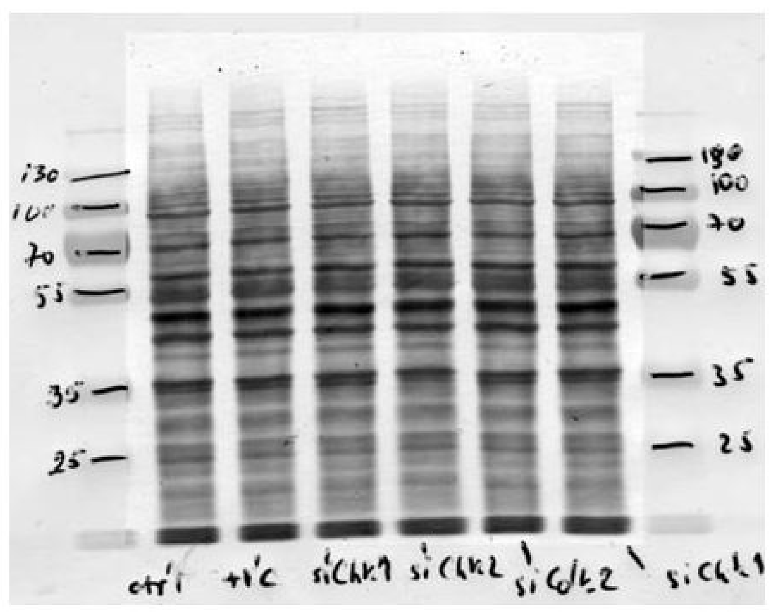

Supplementary Figure S17 continued
